# Superconducting, Topological, and Transport Properties of Kagome Metals CsTi_3_Bi_5_ and RbTi_3_Bi_5_

**DOI:** 10.34133/research.0238

**Published:** 2023-10-02

**Authors:** Xin-Wei Yi, Zheng-Wei Liao, Jing-Yang You, Bo Gu, Gang Su

**Affiliations:** ^1^ School of Physical Sciences, University of Chinese Academy of Sciences, Beijing 100049, China.; ^2^ Department of Physics, Faculty of Science, National University of Singapore, 117551, Singapore.; ^3^ Kavli Institute for Theoretical Sciences, and CAS Center for Excellence in Topological Quantum Computation, University of Chinese Academy of Sciences, Beijing 100190, China.

## Abstract

The recently discovered ATi_3_Bi_5_ (A=Cs, Rb) exhibit intriguing quantum phenomena including superconductivity, electronic nematicity, and abundant topological states. ATi_3_Bi_5_ present promising platforms for studying kagome superconductivity, band topology, and charge orders in parallel with AV_3_Sb_5_. In this work, we comprehensively analyze various properties of ATi_3_Bi_5_ covering superconductivity under pressure and doping, band topology under pressure, thermal conductivity, heat capacity, electrical resistance, and spin Hall conductivity (SHC) using first-principles calculations. Calculated superconducting transition temperature (*T*_c_) of CsTi_3_Bi_5_ and RbTi_3_Bi_5_ at ambient pressure are about 1.85 and 1.92 K. When subject to pressure, *T*_c_ of CsTi_3_Bi_5_ exhibits a special valley and dome shape, which arises from quasi-two-dimensional compression to three-dimensional isotropic compression within the context of an overall decreasing trend. Furthermore, *T*_c_ of RbTi_3_Bi_5_ can be effectively enhanced up to 3.09 K by tuning the kagome van Hove singularities (VHSs) and flat band through doping. Pressures can also induce abundant topological surface states at the Fermi energy (*E*_F_) and tune VHSs across *E*_F_. Additionally, our transport calculations are in excellent agreement with recent experiments, confirming the absence of charge density wave. Notably, SHC of CsTi_3_Bi_5_ can reach up to 226*ℏ* ·(e· Ω ·cm)^–1^ at *E*_F_. Our work provides a timely and detailed analysis of the rich physical properties for ATi_3_Bi_5_, offering valuable insights for further experimental verifications and investigations in this field.

## Introduction

The kagome lattice composed of tiled corner-sharing triangles is a well-known two-dimensional (2D) prototype lattice, in which topological magnetism, superconductivity, and frustration have been extensively studied in recent decades [1,2]. The ideal electronic band structure of kagome lattice is characterized by flat bands, Dirac cones, quadratic band touching points, and van Hove singularities (VHSs) (Fig. S1B). The flat bands originating from the destructive interference of wave functions suggest a strong correlation between electrons and can induce special negative magnetism [3], fractional quantum Hall effect [4,5], Wigner crystallization [6], high-temperature superconductivity [7], etc. Moreover, the spin-orbit coupling (SOC) at the massless Dirac points and quadratic band touching points introduces energy gaps, which can give rise to nontrivial Z_2_ topology akin to topological insulators. These gaps can even exhibit Chern gaps when subjected to magnetization [8,9]. Fermi surface instabilities due to sublattice interference near VHS may cause different long-range charge orders and unconventional superconductivity [10–12]. Additionally, other novel physical properties, including Wely points [13–15], giant anomalous Hall effect [16–18], quantum anomalous Hall effect [19,20], and topological superconductors [21,22], are all intriguing research topics of kagome structure.

The recently discovered AV_3_Sb_5_ (A=K, Rb, Cs) kagome family exhibits various attractive properties [23]. As the pioneering examples of quasi-2D kagome superconductors, their superconducting transition temperatures (*T*_c_) are about 1.8 to 2.5 K under ambient pressure [24–27]. Alongside the emergence of 2 × 2 charge density wave (CDW) induced by VHSs with transition temperatures of about 100 K [28–30], AV_3_Sb_5_ hosts a variety of unconventional charge orders, including a nematic phase reminiscent of iron-based superconductors [31,32], a pair density wave similar to copper-based superconductors [27], and a 4 × 1 CDW that induces striking phase fluctuation [33,34]. Pressure and doping are two effective ways to manipulate the phase diagram of AV_3_Sb_5_. Multiple superconductivity domes simultaneously emerge intertwined with several CDW orders under pressure [30,32,35,36]. Experimental substitution doping with Ti, Nb, Ta, Mo, Mn, Cr, Sn, and As in AV_3_Sb_5_ has been achieved, providing control over superconductivity, CDW, and nematicity [37–46]. CsV_3_Sb_5_, in particular, possesses both abundant nontrivial Z_2_ topological surface states (TSS) and superconductivity [25,47] and has been instrumental in resolving possible Majorana zero modes within vortex cores [48].

High-throughput density functional theory calculations have been employed to analyze a range of compounds based on the AV_3_Sb_5_ prototype structure, revealing 24 dynamically stable compounds with superconductivity, abundant TSS and CDW [49]. Among them, ATi_3_Bi_5_ (A=Rb, Cs) compounds with enhanced *T*_c_ of about 4.8 K and electronic nematicity have recently been synthesized [50–53]. Another kagome superconductor family, similar to ATi_3_Bi_5_, has also been predicted and awaits experimental verification [54]. The nematic order in CsV_3_Sb_5_ is considered as the vestige order of CDW [31]. However, the original lattice structure of ATi_3_Bi_5_ has been confirmed to be highly stable and devoid of CDW, as supported by calculations and thermal transport measurements [49–51,55]. The presence of orbital-selective nematic order and intertwined superconductivity in ATi_3_Bi_5_ provides a new platform for exploring the multiorbital correlation effect in nematic superconductors. In addition, exotic electronic structures have been observed in ATi_3_Bi_5_ using angle-resolved photoemission spectroscopy, including flat band, type II and type III Dirac nodal lines and Z_2_ TSS [56–61]. These fascinating properties make ATi_3_Bi_5_ ideal systems for investigating various kagome-related physics with reference to AV_3_Sb_5_. Consequently, further investigations are imperative, particularly in examining the effects of pressure and doping on the properties of superconductivity and topology.

In this paper, we analyze the superconducting and topological properties of ATi_3_Bi_5_ under pressure and doping by first-principles calculations. Estimated *T*_c_’s of CsTi_3_Bi_5_ and RbTi_3_Bi_5_ at ambient pressure are about 1.85 and 1.92 K. We observe the emergence of a special valley and dome in the *T*_c_ of CsTi_3_Bi_5_ under pressure, which is due to the transition from quasi-2D compression to three-dimensional (3D) isotropic compression within the range of 10 to 20 GPa, accompanied by a decreased background effect across all pressure ranges. Moreover, electron–phonon coupling (EPC) calculations with both a rigid band model and atomic substitution doping confirm the substantial enhancement of superconductivity in RbTi_3_Bi_5_ under doping. Additionally, pressure can lead to the presence of abundant TSS near *E*_F_. We also discuss properties such as thermal conductivity, heat capacity, and electrical resistance, which are consistent with recent experimental findings, indicating the absence of CDW. We find that CsTi_3_Bi_5_ exhibits an intrinsic spin Hall conductivity (SHC) of up to 226*ℏ* ·(e· Ω ·cm)^–1^ at the Fermi energy (*E*_F_). Our findings indicate that both doping and pressure prove to be effective means of tuning the electronic band structures and superconductivity of kagome ATi_3_Bi_5_.

## Results and Discussion

### Electronic structure of CsTi_3_Bi_5_

**Fig. 1.**
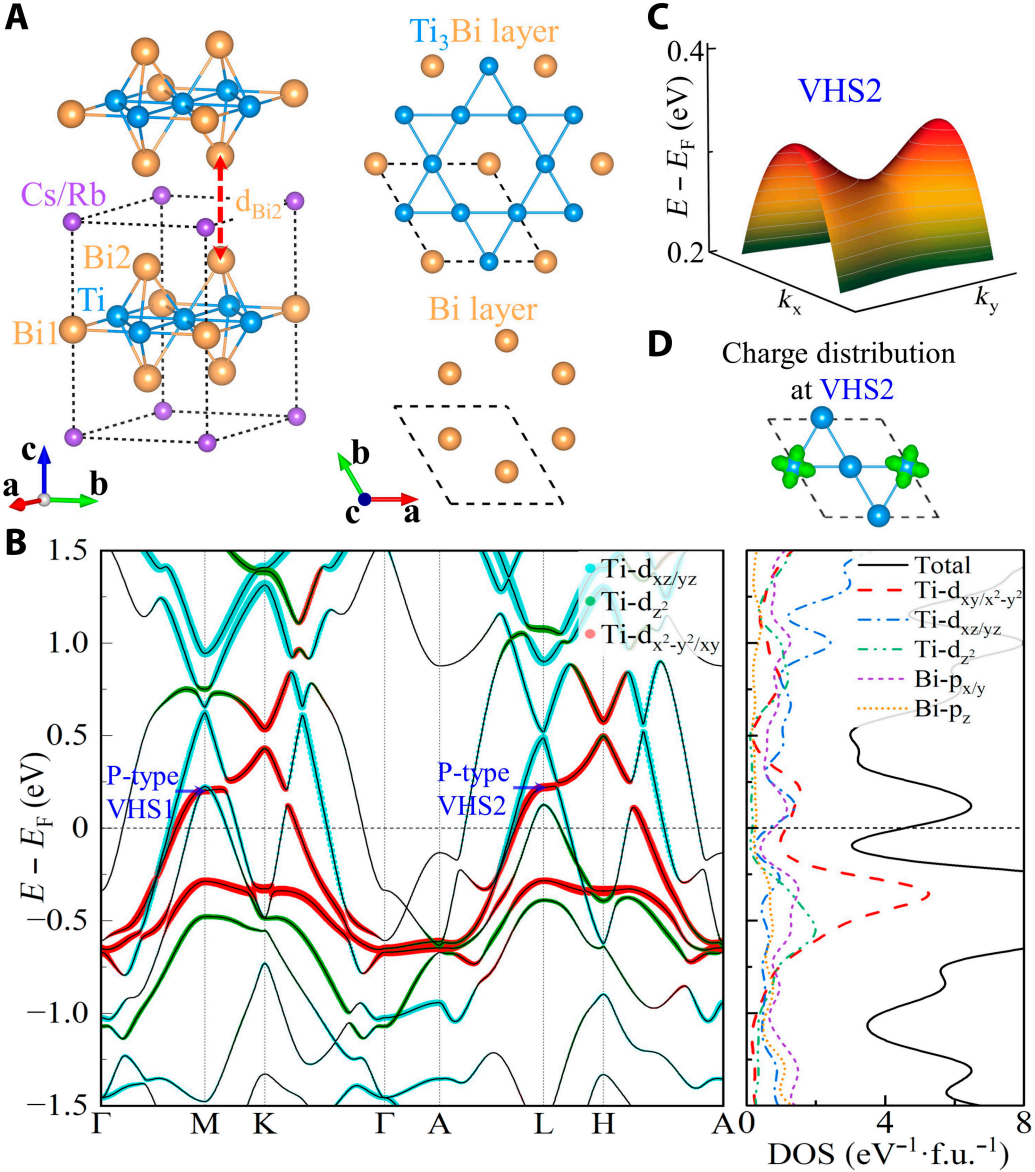
The crystal structure and electronic band structure of CsTi_3_Bi_5_. (A) The side view of crystal structure and top views of Ti_3_Bi kagome layer and Bi hexagonal layer. “d_Bi2_” represents the vertical distance between Bi2 atoms in the adjacent primitive lattices, indicated by the black dotted lines. (B) Calculated electronic band structure projected by different orbitals of Ti atoms and partial DOS with SOC for CsTi_3_Bi_5_. Two kagome VHSs near the Fermi level are labeled as “VHS1” and “VHS2”, respectively. (C) 3D band structure near VHS2, with gray curves indicating constant energy contours. (D) Charge density distribution of the electronic state at VHS2 (isovalue: 0.003 e/bohr^3^).

The ATi_3_Bi_5_ compounds exhibit a layered structure with the space group P6/mmm (No. 191) as illustrated in Fig. 1A. The central Ti_3_Bi layer consists of a Ti-kagome net intertwined with a Bi triangular net, sandwiched between two Bi honeycomb layers. The triangular layers composed of A atoms are loosely bonded with the central Ti–Bi sheets. Bi atoms occupy two distinct Wyckoff positions, designated as Bi1 and Bi2, respectively. The calculated electronic band structures and partial electronic density of states (DOS) with SOC for CsTi_3_Bi_5_ are plotted in Fig. 1B. By assigning colors to the bands based on the weights of different Ti atomic orbitals, we can identify a clear kagome flat band at about −0.4eV, VHSs at M and L points (namely, VHS1 and VHS2) at about 0.2 eV and massive Dirac points at K and H points contributed by Ti-$d_{x^{2}-y^{2}/xy}$ orbitals. The VHSs and flat band of Ti-$d_{x^{2}-y^{2}/xy}$ orbitals give rise to two DOS peaks at the corresponding energy levels. Notably, clear saddle features are observed in the 3D band structure near VHS2 as plotted in Fig. 1C.

Two distinct VHSs arise from the general kagome electronic bands, namely, p-type and m-type VHSs, corresponding to pure and mixed sublattice characteristics at the VHS, respectively. The charge density distribution of the electronic state at the VHS2 shows pronounced in-plane d-orbital features and a p-type character as illustrated in Fig. 1D. Unlike CsV_3_Sb_5_, where all p-type VHSs are located above corresponding Dirac points, the p-type VHS1 and VHS2 of CsTi_3_Bi_5_ lie below Dirac points in Fig. 1B. To clarify this, we construct a tight-binding kagome model with a single orbital on each sublattice and nearest-neighboring (NN) hopping, a three-band Hamiltonian can be written as follows,

$$H=\sum_{\underset{i\neq j}{i,j}}2t\text{cos}\left(k\cdot d_{\mathrm{ij}}\right)c_{i,k}^{†}c_{j,k},\;\left(i,j=A,B,C\right)$$ (1)

where *t*, *d_ij_*, and *k* = (*k_x_*, *k_y_*, *k_z_*) represent the NN hopping parameter, NN space vector of sublattice *i* and *j*, and wavevector of reciprocal space, respectively. *A*, *B*, and *C* represent three kagome sublattices. This toy kagome model produces the flat band spanning the entire Brillouin zone, VHSs at $\bar{M}$, Dirac point at $\bar{K}$, and quadratic band touching point at $\bar{\Gamma }$ as shown in Fig. S1B of the supplemental information. By adjusting *t*, it becomes apparent that for *t* > 0, the p-type and m-type VHSs are located above and below the Dirac points, respectively, and the flat band resides at the top of band structure. Conversely, for *t* < 0, the bands are reversed. This observation indicates that in the kagome sublattice of CsV_3_Sb_5_, all the relevant NN hopping parameters are positive, while for the Ti-d_x²-y²/xy_ orbitals in CsTi_3_Bi_5_, the NN hopping parameters exhibit negative values. P-type VHSs can induce Fermi surface instabilities [10–12], and further investigations are warranted to explore their connection with the nematic and superconducting properties in CsTi_3_Bi_5_.

### Transport properties of CsTi_3_Bi_5_

**Fig. 2.**
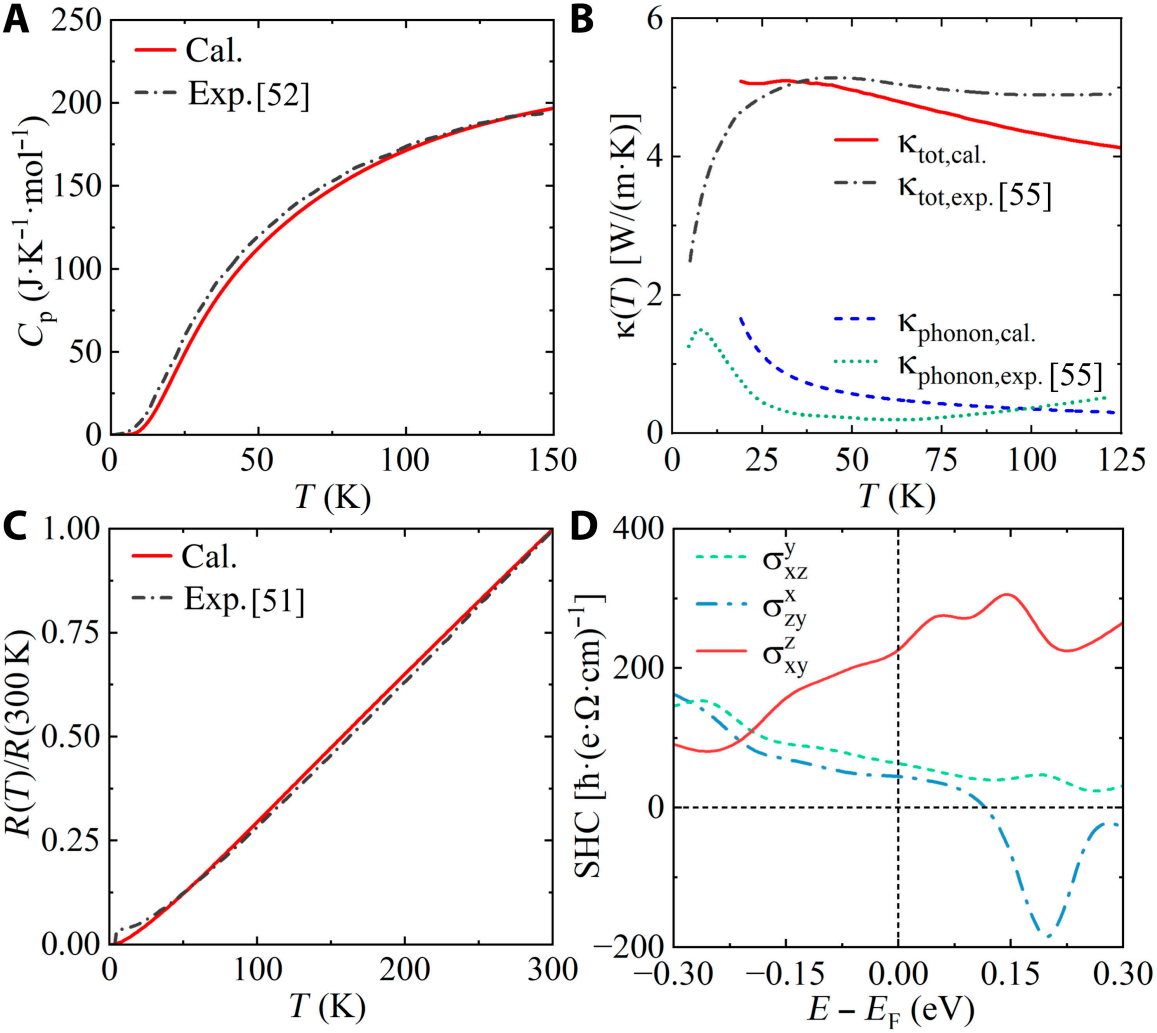
Heat capacity and transport properties of CsTi_3_*Bi*_5_. Calculated temperature dependences of (A) heat capacity, (B) longitudinal thermal conductivity, and (C) longitudinal electrical resistivity, compared with experimentals [51,52,55]. (D) Calculated three independent components of SHC tensor as a function of the chemical potential.

To give a reference for the transport properties of ATi_3_Bi_5_, we calculate the temperature dependence of heat capacity *C*_p_, thermal conductivity *κ*, and resistance *R* for CsTi_3_Bi_5_. These results are compared with recent experimental data as plotted in Fig. 2A to C. Part of the calculated results for RbTi_3_Bi_5_ are listed in Fig. S3. Overall, our transport calculations are in quantitative agreement with the experimental findings, demonstrating the reliability of our calculations. Obviously, there are no phase transition signatures in any of the transported quantities, suggesting the absence of CDW. In contrast to CsV_3_Sb_5_, the total thermal conductivity *κ*_tot,cal_ of CsTi_3_Bi_5_ is primarily governed by electronic contributions rather than phonon thermal conductivity *κ*_phonon,cal_ [55]. We choose the results with the highest residual resistance ratios from [51], where the impurity concentration is the lowest in that experiment. The resistance of CsTi_3_Bi_5_, as shown in Fig. S3B, is much smaller than that of AV_3_Sb_5_ [25]. The calculated *R*(*T*)/*R*(300 K) values align well with the experimental results at high temperatures but underestimate the experimental data below 30 K as shown in Fig. 2C. This discrepancy arises that our resistance calculations solely consider the electron–phonon interaction and do not account for electron–electron interactions. In the experimental resistance–temperature behavior, a quadratic dependence below 30 K and a linear dependence above 50 K can be observed, corresponding to electron–electron and electron–phonon interactions in the normal metal, respectively. It is important to note that our calculations of the transport properties are performed on the normal phase of CsTi_3_Bi_5_ and not the superconducting phase. Therefore, our calculations do not capture the information regarding the superconducting phase transition.

In addition to the aforementioned transport properties, we also calculate the three independent components of SHC tensor, i.e., $\sigma_{\mathrm{xy}}^{z}$, $\sigma_{zy}^{x}$, and $\sigma_{\mathrm{xz}}^{y}$, as a function of the chemical potential for CsTi_3_Bi_5_ in Fig.2D. The magnitudes of three components can reach up to 226*ℏ* ·(e· Ω· cm)^–1^ at *E*_F_. The large SHC comes from a narrow band gap opened by large SOC [62]. The large SHC observed in CsTi_3_Bi_5_ holds great potential for applications in spintronic devices.

### Superconductivity of CsTi_3_Bi_5_ under pressure

The superconducting *T*_c_ of CsV_3_Sb_5_ at pressures above 2 GPa, in the absence of CDW, have been found to be in good agreement between EPC calculations and experimental results [63,64]. Therefore, we can also expect density functional theory calculations to accurately capture the true superconductivity in ATi_3_Bi_5_. The estimated *T*_c_’s of CsTi_3_Bi_5_ and RbTi_3_Bi_5_ at ambient pressure are about 1.85 and 1.92 K, respectively. The superconducting properties of both materials share remarkable similarities, so we select CsTi_3_Bi_5_ for a detailed analysis of superconductivity under pressure.

**Fig. 3.**
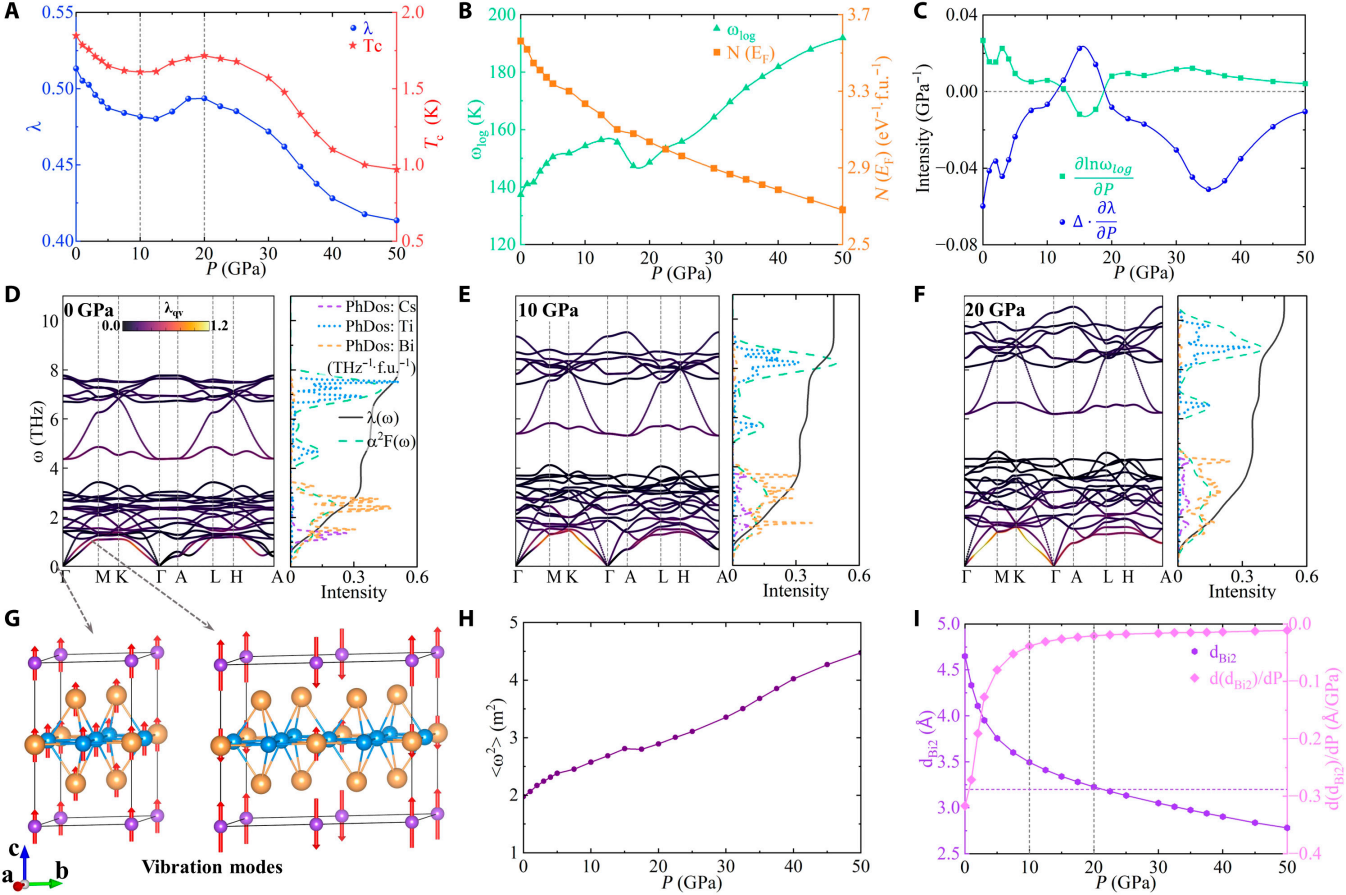
Superconducting properties of CsTi_3_Bi_5_ under pressure in the range of 0 to 50 GPa. (A) Superconducting transition temperature *T*_c_ and EPC *λ* under pressure. (B) Logarithmic average frequency *ω*_log_(K) and electronic DOS per formula unit (f.u.) at the Fermi energy *N*(*E*_F_) under pressure. (C) $\frac{\partial \text{ln}\omega_{\text{log}}}{\partial P}$ and $\Delta \cdot \frac{\partial \lambda }{\partial P}$ under pressure. Phonon spectra colored by EPC strength *λ*_qν_, projected phDOS, Eliashberg spectral function *α*^2^*F*(*ω*), and cumulative frequency-dependent EPC *λ*(*ω*) at (D) 0, (E) 10, and (F) 20 GPa, respectively. (G) Vibration modes at the lowest acoustic branch near Γ and M points, which both are contributed by the vibrations along the c axis. (H) The average of square of phonon frequency ⟨*ω*^2^⟩ under pressure. (I) d_Bi2_ denoted in Fig. 1A and *d*(*d*_Bi2_)/*dP* under pressure. The horizontal dashed line indicates the Bi atomic diameter of 3.2Å.

In previous reports on AV_3_Sb_5_ (A = K, Rb, Cs) pressurized experiments, the upper pressure limit was typically applied below 50 GPa [28,30,32,35,36,65]. Our calculation results show that some physical quantities related to superconductivity change monotonically with pressure above 20 GPa. Thus, we choose the pressure range of 0 to 50 GPa to investigate superconducting properties in the main text. The additional results at the pressure of 50 to 150 GPa can be found in Fig. S4. Our calculated *T*_c_ and EPC constant *λ* as a function of pressure are shown in Fig. 3A. Both parameters decrease in the ranges of 0 to 10 and 20 to 50 GPa, while they increase within the range of 10 to 20 GPa, forming a unique valley and dome shape under pressure. The corresponding logarithmic average of the phonon frequency *ω_log_* and the electronic DOS at the Fermi energy *N*(*E*_F_) are shown in Fig. 3B. *N*(*E*_F_) shows an almost linear decrease with increasing pressure, while *ω_log_* exhibits an overall increase but with a decreasing trend specifically within the 10 to 20 GPa. It is noted that *T*_c_ is explicitly proportional to *ω_log_* as described by McMillan semi-empirical formula [66,67], $T_{c}=\frac{\omega_{\log}}{1.2}\text{exp}\left[-\frac{1.04\left(1+\lambda \right)}{\lambda -\mu^{*}\left(1+0.62\lambda \right)}\right]$, whereas the variation of *T*_c_ in CsTi_3_Bi_5_ shows an opposite trend compared with *ω_log_* in Fig. 3A and B. To address this apparent contradiction, we differentiate McMillan formula with respect to pressure value (*P*), which gives

$$\begin{array}{ll} & \frac{dT_{c}}{\mathrm{dP}}=T_{c}\cdot \left[\frac{\partial \text{ln}\omega_{\text{log}}}{\partial P}+\Delta \cdot \frac{\partial \lambda }{\partial P}\right],\\ \text{and} & \Delta =\frac{1.04\left(1+0.38\mu^{*}\right)}{{\left[\lambda -\mu^{*}\left(1+0.62\lambda \right)\right]}^{2}}. \end{array}$$ (2)

We calculate two terms in the brackets separately and illustrate them in Fig. 3C. It becomes evident that *λ* and *ω_log_* make totally opposite contributions to $\frac{dT_{c}}{\mathrm{dP}}$ across all pressure ranges. However, $\frac{\partial \text{ln}\omega_{\text{log}}}{\partial P}$ is much smaller than $\Delta \cdot \frac{\partial \lambda }{\partial P}$. This observation explains the reason why *T*_c_ follows the same trend as *λ* but not *ω_log_* under pressure.

The remaining question is why *λ* (*T*_c_) displays a unique valley and dome shape under pressure. To shed light on this, we present the phonon spectra, projected phonon DOS (phDOS), Eliashberg spectral function *α*^2^*F*(*ω*), and cumulative frequency-dependent EPC *λ*(*ω*) in Fig. 3D to F. The corresponding results for other pressures can be found in Fig. S5. As pressure increases, the phonon frequencies (*ω*) within the phonon spectra noticeably increase, which is a common characteristic observed in many materials. This makes the average of the square of phonon frequency ⟨*ω*^2^⟩ almost linearly increase under pressure as seen in Fig. 3H. From the definition of *λ*, we have $\lambda =\frac{N\left(E_{F}\right)\langle I^{2}\rangle }{M\langle \omega^{2}\rangle }$ and $\langle \omega^{2}\rangle =\left[\frac{2}{\lambda }\int_{0}^{\infty }‍\alpha^{2}F\left(\omega \right)\mathrm{\omega d\omega}\right]$ ,where ⟨*I*^2^⟩ is the mean-square electron-ion matrix element, and *M* is the ionic mass. It is clear that *λ* is proportional to *N*(*E*_F_) and inversely proportional to ⟨*ω*^2^⟩. The combination of *N*(*E*_F_) and ⟨*ω*^2^⟩ results in an overall decrease in *λ*. This mechanism accounts for the downward trend of *λ* and *T*_c_ within the range of 0 to 10 and 20 to 50 GPa.

Upon conducting a more detailed analysis of the phonon spectra and phDOS, we can find that the phonon spectra under pressure can be well separated into distinct high- and low-frequency regions. Specifically, the high-frequency region is mainly contributed by kagome Ti atoms, displaying clear kagome phonon flat band and VHSs. On the other hand, the low-frequency region primarily corresponds to the vibrational modes of Cs and Bi atoms. By separating *λ* into two components, namely, *λ*_Cs+Bi_ and *λ*_Ti_, we observe that *λ*_Ti_ gradually decreases with increasing pressure due to the aforementioned background effect, while *λ*_Cs+Bi_ exhibits exactly the same trend as *λ*, as seen in Fig. S6A. Consequently, our analysis focuses on examining the low-frequency region within the phonon spectra in order to elucidate the trend of *λ*.

The total EPC *λ* can be decomposed into the average coupling strengths *λ*_**q***ν*_ over the Brillouin zone with *λ* = ∑_**q**,*ν*_ ‍ *λ*_**q***ν*_. By coloring the phonon spectra with *λ_qv_*, we find that the high *λ*_**q***ν*_ are mainly concentrated in the lowest acoustic branch. Taking vibration modes of the lowest acoustic branch near Γ and M point as examples in Fig. 3G, we observe that these modes primarily involve vibrations along the c axis. Thus, based on our calculations, we can infer that the anomalous increase of *λ* within 10 to 20 GPa originates from the contributions of *λ*_**q***ν*_ associated with the out-of-plane atomic vibrations.

Next, we analyze superconductivity under pressure from a structural perspective. The vertical distance between Bi2 atoms in adjacent Ti_3_Bi_5_ sheets is defined as d _Bi2_ as illustrated in Fig. 1A. Because of the quasi-2D structural features of CsTi_3_Bi_5_, d_Bi2_ is quite large, and interlayer interactions are weak. At low pressures, *d*(*d*_Bi2_)/*dP* exhibits large values and changes dramatically. However, as the pressure exceeds 10 GPa, the rate of change in d_Bi2_ starts to slow down. Furthermore, when *P* > 20 GPa, it approaches a nearly constant value. This trend is also reflected in the ratio of the out-of-plane and in-of-plane lattice parameters *c*/*a* as depicted in Fig. S6B. At 20 GPa, d_Bi2_ becomes approximately equal to the Bi atomic diameter of 3.2Å, indicating the formation of covalent bonds among the Bi2 atoms around this pressure. These results demonstrate that the structure undergoes a quasi-2D compression below 10 GPa. The weak coupling between Cs atoms and Ti_3_Bi_5_ sheets leads to a pronounced response of the out-of-plane lattice parameter *c* to applied pressure. At 10 GPa, the Bi atoms of neighboring Ti_3_Bi_5_ sheets start to interact, leading to a dramatic enhancement of *λ*_**q***ν*_ associated with out-of-plane vibrations. However, as the pressure exceeds 20 GPa, the structure exhibits a 3D isotropic compression characteristic. The interaction between Bi2 atoms no longer increases but gradually decreases due to the underlying background effect mentioned earlier.

### Topological states of CsTi_3_Bi_5_ under pressure

**Fig. 4.**
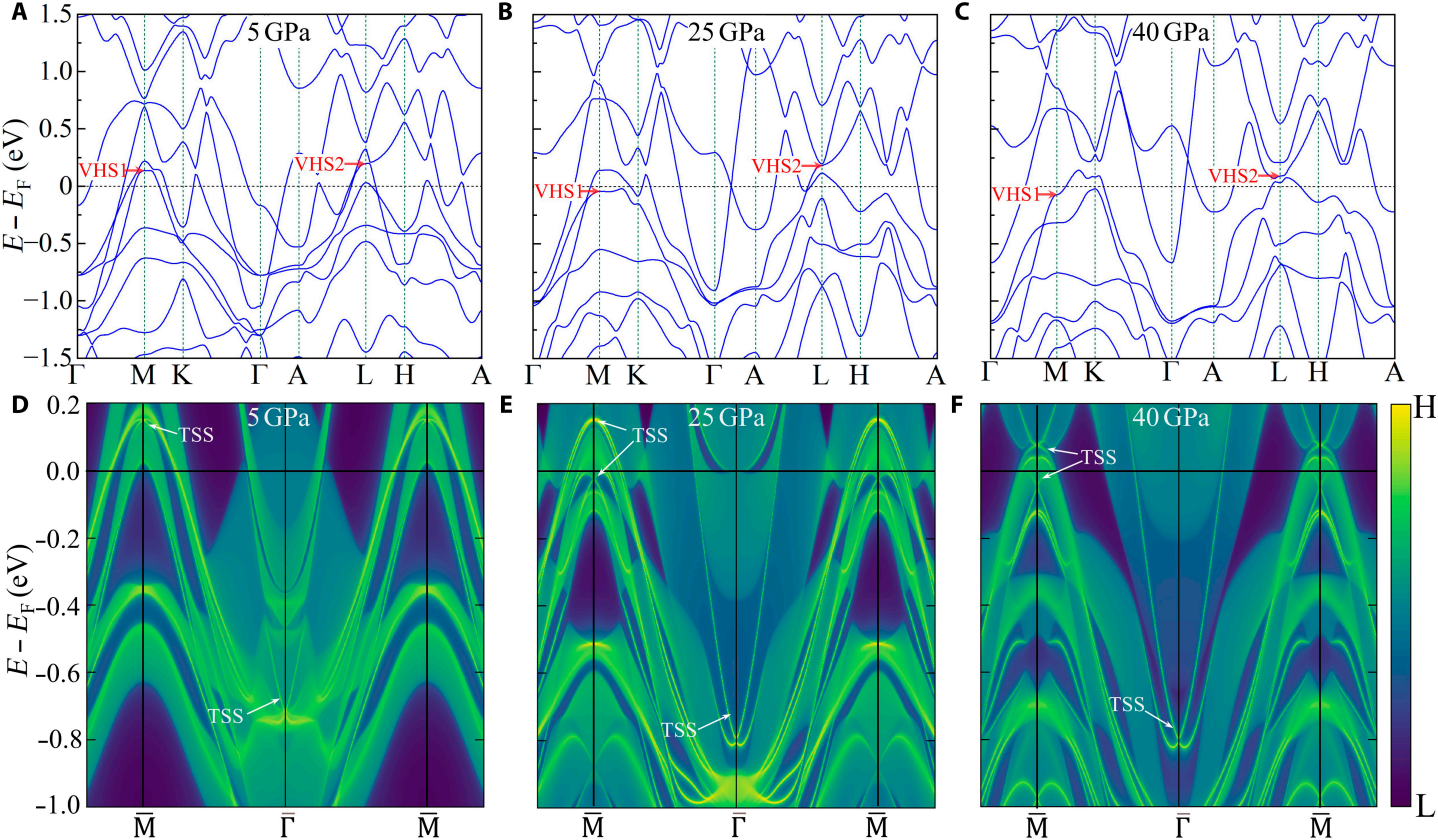
Electronic band structures and surface-state spectra under pressure for CsTi_3_Bi_5_. Electronic band structures with SOC at (A) 5, (B) 25, and (C) 40 GPa. Surface-state spectra along $\bar{M}-\bar{\Gamma }-\bar{M}$ paths on the (001) plane at (D) 5, (E) 25, and (F) 40 GPa. TSS are indicated with white arrows.

Next, we explore the effect of pressure on electronic structures. We illustrate the calculation results at pressures of 5, 25, and 40 GPa in Fig. 4A to C, which depict the electronic band structures of CsTi_3_Bi_5_ with SOC. The increased interlayer interaction under pressure profoundly impacts the band structures, leading to notable changes in the energy levels of VHSs and flat band, particularly at higher pressures. With increasing pressure, both VHSs and flat band gradually shift downwards. Remarkably, the VHSs progressively approach the vicinity of the Fermi level under pressure. Exploring the potential nematicity and other phases induced by VHSs through pressure engineering holds substantial interest.

The nontrivial TSS near Γ point of CsTi_3_Bi_5_ have been experimentally detected [51,57,58]. However, these TSS are approximately 0.8 eV below the Fermi level, limiting their substantial effects on transport and other related properties. By examining the surface-state spectra of CsTi_3_Bi_5_ under pressure in Fig. 4D to F, we observe the emergence of multiple TSS at time-reversal invariant momenta $\bar{M}$ and $\bar{\Gamma }$, many of which are located in proximity to the Fermi level. Under pressure, these TSS undergo slight energy shifts. Importantly, once these TSSs emerge at a specific pressure, further increases in pressure do not eliminate them. The TSS in CsTi_3_Bi_5_ exhibits high tunability and sensitivity to pressure, primarily due to the dramatic changes in electronic structures.

### Superconductivity of RbTi_3_Bi_5_ under doping

RbTi_3_Bi_3_ has similar electronic structure and phonon spectrum to CsTi_3_Bi_3_ as shown in Fig. S7. Therefore, we can expect RbTi_3_Bi_3_ and CsTi_3_Bi_3_ to exhibit similar topological, transport, and superconducting properties. As a result, we do not replicate the calculations performed above for RbTi_3_Bi_5_. Since the *T*_c_ of RbTi_3_Bi_5_ is slightly higher than that of CsTi_3_Bi_5_ in our calculations, in this section, we focus on a comprehensive investigation of superconductivity under doping using RbTi_3_Bi_5_.

**Fig. 5.**
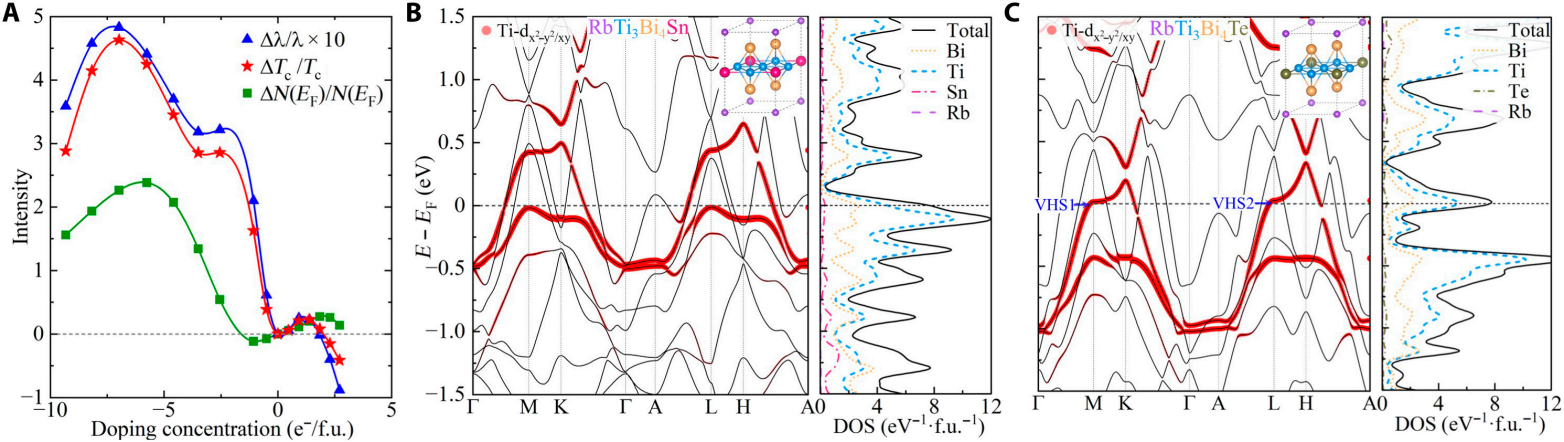
Superconductivity and electronic structures of RbTi_3_Bi_5_ under doping. (A) Relative changes of *λ*, *T*_c_, and N(E_F_) under doping using the ragid band model: Δ*λ*/*λ*, Δ*T*_c_/*T_c_*, and Δ*N*(*E*_F_)/*N*(*E*_F_). Calculated electronic band structures projected by Ti-d _x2−y2/xy_ orbitals and partial DOS with SOC for (B) RbTi_3_Bi_4_Sn and (C) RbTi_3_Bi_4_Te, where the insets show the doped structures. Two kagome VHSs near the Fermi level of RbTi_3_Bi_4_Te are labeled as “VHS1” and “VHS2”, respectively.

Doping is an effective method to tune electronic structures and superconductivity. Extensive studies have successfully achieved doping in AV_3_Sb_5_ to manipulate the competition between superconductivity, CDW, and nematicity [37–46]. Notably, the concentration of Sb replacement doping with Sn in AV_3_Sb_5_ can reach up to 30% [40]. With reference to AV_3_Sb_5_, doping in ATi_3_Bi_5_ is highly feasible. We first employ a rigid band model to analyze the effect of electron and hole doping on superconductivity. Figure 5A illustrates the relative changes in *λ*, *T_c_*, and *N*(*E*_F_) under doping. Both electron and hole doping result in enhancements of *N*(*E*_F_) due to the movement of VHSs and flat band toward the Fermi level. As a consequence, there are dramatic improvements in *λ* and *T*_c_. Especially under hole doping, the highest *N*(*E*_F_) can be increased by nearly 2.5 times, and *T*_c_ can be increased by nearly 5 times.

In addition to the rigid band model, we also explore specific element substitution doping. Alkali metal substitutions of ATi_3_Sb_5_, including LiTi_3_Bi_5_, NaTi_3_Bi_5_, and KTi_3_Bi_5_, are found to be unstable according to our previous study [49]. Instead, we substitute one Ti or Bi atom in each unit cell of ATi_3_Bi_5_, corresponding to 33% Ti or 20% Bi substitution. Ti is replaced by its neighboring elements (Sc, V, Y, Zr, and Nb), while Bi is substituted by Ga, Sn, Sb, Te, Pb, and Bi. As illustrated in Fig. 1A, the Bi atoms occupy two Wyckoff positions that need to be distinguished. Taking Te doping as an example, we denote the substitution of Bi2 as RbTi_3_TeBi_4_, and the substitution of Bi1 as RbTi_3_Bi_4_Te as shown in Fig. S8A. For all doped structures, we first carry out fully geometric relaxation and self-consistent iterations in nonmagnetic, ferromagnetic, and antiferromagnetic configurations as seen in Fig. S8B and C. The energies of the three magnetic configurations of all structures are listed in Table S1, which determines the ground state magnetic configuration. Among the doped structures with Bi substitution, the energy of the replacement of Bi2 is consistently higher than that of Bi1. This suggests that the impurity prefers to substitute the Wyckoff position of Bi1. In addition, Sc-, Y-, and Ga-doped CsTi_3_Bi_5_ and RbTi_3_Bi_5_ all exhibit ferromagnetism, which suppresses superconductivity. Therefore, these structures are not considered in the subsequent analysis.

For these doped structures, we calculate their superconductivity as summarized in Table. Several doped structures show varying degrees of improvement compared to the original phase’s *T*_c_. *T*_c_ of RbTi_3_Bi_4_Sn and RbTi _3_Bi_4_Pb belonging to hole doping can be raised to around 3 K. However, when increasing the doping concentration to 40% for Sn and Pb doping, denoted as RbTi_3_BiSn_2_ and RbTi_3_Bi_3_Pb_2_, the results indicate that there is no obvious increase in *T*_c_’s. The electronic bands and DOS of these doped structures are given in Figs. S9 to S24. The kagome VHSs and flat band originating from Ti in-plane orbitals in the band structures are prominent, which undergo dramatic changes upon Ti doping but relatively maintain structural characteristics in the cases of Bi doping. As examples of hole and electron doping, respectively, the electronic energy bands and DOS of RbTi_3_Bi_4_Sn and RbTi_3_Bi_4_Te are plotted in Fig. 5B and C. The kagome flat band of RbTi_3_Bi_4_Sn and the VHSs of RbTi_3_Bi_4_Te are shifted close to the Fermi level, and the DOS originating from the flat band and VHSs exhibit pronounced peaks near the Fermi level. This indicates a shift of the Fermi level while maintaining the character of kagome band structures. These results are consistent with the above rigid band model, demonstrating that both electron and hole doping can improve *T*_c_ by tuning the kagome electronic structures and increasing the DOS through doping. The effective modulation of superconducting properties and kagome electronic structures by doping highlights the need for further experimental investigations in this system.

**Table.** Superconductivity of doped structures for RbTi_3_Bi_5_. EPC λ(ω = ∞), logarithmic average frequency *ω*_log_ (K), and *T*_c_ of doped structures.

	RbTi_3_Bi_5_	RbVTi_2_Bi_5_	RbZrTi_2_Bi_5_	RbNbTi_2_Bi_5_	RbTi_3_Bi_4_Te	RbTi_3_Bi_4_Sb	RbTi_3_Bi_4_Sn	RbTi_3_Bi_4_Pb	RbTi_3_Bi_3_Sn_2_	RbTi_3_Bi_3_Pb_2_
*λ*	0.520	0.578	0.525	0.586	0.526	0.513	0.605	0.608	0.612	0.622
*ω_log_*(K)	135.8	131.3	113.7	126.5	137.7	144.9	124.6	117.8	127.6	119.1
*T*_c_(K)	1.92	2.67	2.49	2.69	2.04	1.94	2.92	2.81	3.09	3.03

## Discussion

The two superconducting domes observed in AV_3_Sb_5_ at low pressures are commonly believed to be related to the competition of CDW phases [30,32,36]. However, theoretical analyses of the superconducting dome at around 20 to 40 GPa are limited [65,68,69]. The properties of CsTi_3_Bi_5_ and AV_3_Sb_5_ at high pressure, in the absence of CDW, are similar, suggesting that our explanations for the superconducting dome and valley in CsTi_3_Bi_5_ may also apply to AV_3_Sb_5_. Recent research attributes the appearance of high-pressure superconducting dome in AV_3_Sb_5_ to transitions from hexagonal to monoclinic structures [70]. In contrast, our work demonstrates that the appearance of a similar dome in CsTi_3_Bi_5_ does not rely on structural phase transitions but rather originates from the transition from quasi-2D to 3D compression. Further investigations are needed to explore the similarities and differences in the superconducting properties of ATi_3_Bi_5_ and AV_3_Sb_5_ under pressure.

Exploring the possible phase transitions associated with structure or electronic orders in CsV_3_Sb_5_ is an intriguing topic [30,32,36,70]. Phonon spectra of ATi_3_Bi_5_ without imaginary frequencies in the pressure of 0 to 50 GPa in Fig. 3D to F and Figs. S3 and S5B show good dynamic stability, excluding the possibility of structural phase transition under pressure, however, an electronic nematic phase of CsTi_3_Bi_5_ at ambient pressure has been experimentally observed [51,53]. This nematic phase may be suppressed and eventually disappears under pressure, similar to the behaviors of CsV_3_Sb_5_ in experiment [32]. Consequently, there may be a possible nematic phase transition under pressure, which deserves further experimental study.

It should be noted that while *λ*_Ti_ contributes approximately 35% to the total EPC *λ* as shown in Fig. S6A, the electronic states near the Fermi level are primarily dominated by the kagome Ti atoms, as illustrated in Fig. 1B. Calculated electronic DOS projected on different atoms for CsTi_3_Bi_5_ in Fig. S2 indicate that about 64% of the DOS at the Fermi level come from Ti orbitals. In addition, tuning the Fermi level through hole doping can dramatically enhance superconductivity, mainly due to the enhancement of the DOS of Ti atoms as discussed in Superconductivity of RbTi_3_Bi_5_ Under Doping. Therefore, kagome lattice composed by Ti atoms plays a crucial role to the superconductivity.

Fu and Kane [71] proposed that the proximity effect between the helical Dirac TSS of a topological insulator and an s-wave superconductor can induce Majorana zero modes, which can be utilized for topological quantum calculations. This effect has been achieved in CsV_3_Sb_5_ [48]. Based on our calculations, pressure effectively induces abundant Dirac TSS near the Fermi level, whose combination with superconductivity theoretically gives rise to Majorana zero modes. This opens up another fascinating research topic for ATi_3_Bi_5_ and its potential for exploring topological quantum phenomena.

The interlayer van der Waals forces present in ATi_3_Bi_5_ facilitate their mechanical exfoliation from the bulk structures. We explore the structural stability and superconducting properties of CsTi_3_Bi_5_ in the 2D monolayer limit. Three slab structures, namely, CsTi_3_Bi_5_ (slab1), Ti_3_Bi_5_ (slab2), and Cs_2_Ti_3_Bi_5_ (slab3), with different combinations of Ti_3_Bi_5_ layer and Cs layer are considered as seen in Fig. S25. Phonon calculations show that slab2 has minimal imaginary frequencies, while slab1 and slab3 display good dynamic stability. However, superconducting calculations reveal that their *T*_c_’s are nearly equal to that of the bulk structures.

## Conclusion

In summary, we preemptively analyze superconducting and topological properties under pressure and doping for ATi_3_Bi_5_, as well as their thermal conductivity, heat capacity, electrical resistance, and SHC using first-principles calculations. The estimated *T*_c_ of CsTi_3_Bi_5_ presents a special down-up-down trend with increasing pressure. The two decreasing sections can be attributed to the decrease in EPC resulting from reduced electronic DOS and ⟨*ω*^2^⟩ with increasing pressure. Within this overall decreasing trend, the abnormally increased part is associated with the sharply enhanced EPC in the low-frequency region induced by vertical vibration modes, which is related to the transition from anisotropic to isotropic compression from a structural perspective. With rigid band model calculations under doping, *T*_c_ of RbTi_3_Bi_5_ can increase by nearly 5 times accompanied by increased electronic DOS and EPC. Calculations for different atomic substitution structures in RbTi_3_Bi_5_ show improvements of *T*_c_ from 1.9 to 3.1 K. Furthermore, doping and pressure serve as effective means to tune the kagome VHSs and flat band positions close to *E*_F_. Pressure can also induce abundant Dirac-type TSS at *E*_F_. Additionally, our transport calculations agree well with recent experimental results, indicating the absence of CDW. The resistance of CsTi_3_Bi_5_ shows normal metal behavior, and its thermal transport is predominantly governed by electronic contributions. The SHC of CsTi_3_Bi_5_ can reach up to 226*ℏ* ·(e· Ω ·cm)^–1^ near the Fermi level. Our comprehensive analysis of the diverse physical properties in ATi_3_Bi_5_ underscores the need for further experimental exploration in this field.

## Methods

The methods can be found in the Supplementary Materials.

## Data Availability

The data that support the findings of this study are available from the corresponding authors upon reasonable request.

## Supplementary Materials

Supplementary 1 Methods Figs. S1 to S25 Tables S1 and S2 References [72–80]
