# Supplementary material for: Superconducting, Topological, and Transport Properties of Kagome Metals CsTi3Bi5 and RbTi3Bi5
Source: Research (Wash D C). 2023 Oct 2;6:0238. doi: 10.34133/research.0238 (PMC10543885; doi:10.34133/research.0238)
Supplement: Supplementary 1 — Methods Figs. S1 to S25 Tables S1 and S2 References [72–80] [file research.0238.f1.pdf]

6 Supplementary Materials

6.1 Methods

DFT calculations are performed using *Vienna ab initio simulation package* (VASP) [72] and *QUANTUM ESPRESSO* (QE) [73]. The parameters for VASP are set as follows: the convergence criterion of atomic force is less than 1meV/Å; cutoff energy of plane-wave is taken as 520 eV; and total energy convergence threshold is set to 10^{-7} eV/atom. For QE, the convergence criterion of atomic force is less than 10^{-7} Ry/a.u.; kinetic energy cutoff of wavefunctions and charge density are 60 Ry and 600 Ry, respectively; convergence threshold cutoffs of wavefunction and ionic minimization total energy is 10^{-9} Ry; a Γ centered 10×10×6 Monkhorst-Pack k-mesh is used in the self-consistent calculation, and the q-point grid is set to 5×5×3 for EPC calculation. The calculated lattice parameters of $RbTi_3Bi_5$ and $CsTi_3Bi_5$ are a=5.75Å, c=9.36Å and a=5.77Å, c=9.55Å, respectively, agreeing well with earlier experimental reports [51].

First-principle calculations on phonon-mediated superconductors based on BCS theory are carried out by QE [73]. According to the McMillan semi-empirical formula [66, 67] for T_c within the framework of weak-coupling theory, T_c can be obtained by

$$T_c = \frac{\omega_{log}}{1.2}\exp[-\frac{1.04(1+\lambda)}{\lambda-\mu^*(1+0.62\lambda)}]. \tag{3}$$

Here ω_{log} is the logarithmic average of phonon frequency, λ is a dimensionless parameter describing EPC, and μ^* is an empirical parameter describing the Coulomb repulsion. We choose commonly used $\mu^* = 0.10$ for all calculations. ω_{log} and λ can be calculated by

$$\omega_{log} = \exp[\frac{2}{\lambda}\int_0^\infty \frac{d\omega}{\omega}\alpha^2F(\omega)log\omega], \tag{4}$$

and

$$\lambda(\omega) = 2\int_0^\omega \frac{\alpha^2F(\omega)}{\omega}d\omega, \tag{5}$$

respectively, where $\alpha^2F(\omega)$ denotes the Eliashberg electron-phonon spectral function.

We use the *Wannier90* package [74] to fit the Wannier functions, construct effective tight-binding (TB) Hamiltonian, and calculate SHC, where Cs-s, Ti-d and Bi-p orbitals are chosen to be projected.

SHC tensor is calculated by employing the Kubo formula [75, 76]

$$\sigma^\gamma_{\alpha\beta} = e\hbar\int\frac{d\mathbf{k}}{(2\pi)^3}\Omega^\gamma_{\alpha\beta}(\mathbf{k}), \tag{6}$$

where $\Omega^\gamma_{n,\alpha\beta}(\mathbf{k})$ is the spin Berry curvature. The k-mesh of 200×200×200 are adopted for the spin Berry curvature integral, and an extra 5×5×5 fine mesh is added around points with $\Omega^\gamma_{n,\alpha\beta}(\mathbf{k})$ exceeding $100\mathbf{Å}^2$. With the effective TB Hamiltonian, we calculate topological properties using the iterative surface Green's function method with *WannierTools* package [77].

The electronic transport properties and heat capacity are calculated by solving the Boltzmann transport equation with BoltzTrap package[78]. Phonon heat capacity is calculated with *phonopy*

based on quasi harmonic approximation [79]. Electronic resistance is calculated by solving Ziman's resistivity formula with *Electron-phonon Wannier* (EPW) package [80].

6.2 Supplemental Figures

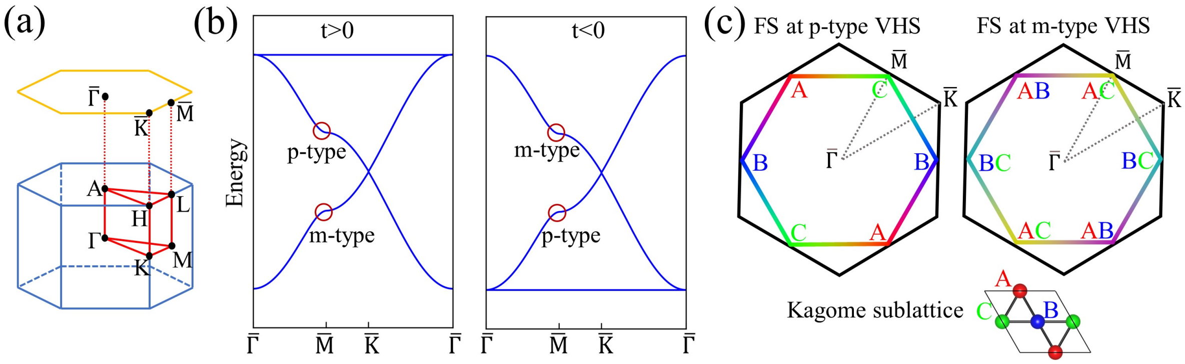

FIG. S1. BZ and band structures of kagome tight-binding (TB) model. (a) 3D and 2D Brillouin zone (BZ) with high symmetry points. 3D and 2D BZ are drawn with blue and orange solid lines, respectively. (b) Band structures of kagome TB model with nearest-neighboring hopping parameter t>0 and t<0, respectively. (c) Fermi Surface (FS) of kagome bands at p-type and m-type van Hove singularities (VHSs). Red, blue and green colors of the FS represent the contributions of three kagome sublattices A, B, and C weights as shown in the bottom panel.

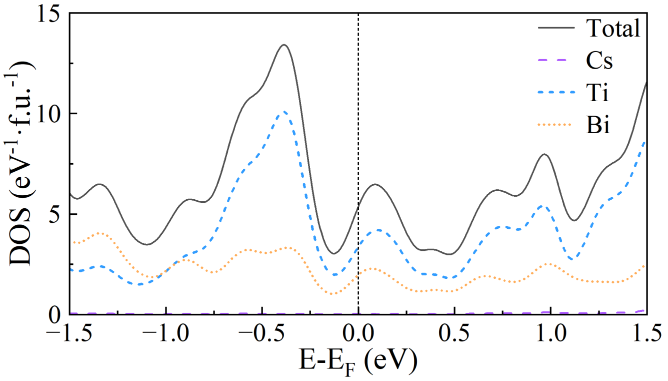

FIG. S2. Calculated electronic density of states projected on different atoms with SOC for $CsTi_3Bi_5$.

TABLE S1. Total energy per formula unit (eV/f.u.) of nonmagnetic (NM), ferromagnetic (FM), and antiferromagnetic (AFM) configurations, total magnetic moment per fomula unit M (μ_B/f.u.) and the ground state for all doped structures.

Structure	E_{NM} (eV/f.u.)	E_{FM} (eV/f.u.)	E_{AFM} (eV/f.u.)	M (μ_B/f.u.)	Ground state
$CsScTi_2Bi_5$	-50.4981	-50.5073	-50.5015	0.545	FM
$CsYTi_2Bi_5$	-50.3004	-50.317	-50.3005	1.071	FM
$CsNbTi_2Bi_5$	-54.0667	-54.0667	-54.0667	0	NM
$CsVTi_2Bi_5$	-52.6233	-52.6202	-52.6205	0	NM
$CsZrTi_2Bi_5$	-52.9164	-52.9164	-52.9164	0	NM
$CsTi_3Bi_4Pb$	-51.3923	-51.3922	-51.3923	0	NM
$CsTi_3PbBi_4$	-51.2919	-51.2919	-51.2919	0	NM
$CsTi_3Bi_4Sb$	-52.4239	-52.4237	-52.4237	0	NM
$CsTi_3SbBi_4$	-52.0766	-52.0766	-52.0766	0	NM
$CsTi_3Bi_4Sn$	-51.5385	-51.5385	-51.5385	0	NM
$CsTi_3SnBi_4$	-51.1367	-51.1367	-51.1367	0	NM
$CsTi_3Bi_4Te$	-51.8413	-51.8413	-51.8413	0	NM
$CsTi_3TeBi_4$	-51.8102	-51.8102	-51.8102	0	NM
$CsTi_3Bi_4Ga$	-50.0249	-50.0621	-50.0217	0.924	FM
$CsTi_3GaBi_4$	-49.8179	-49.8178	-49.8178	0	NM
$RbScTi_2Bi_5$	-50.2444	-50.2534	-50.2476	0.542	FM
$RbYTi_2Bi_5$	-50.0407	-50.0534	-50.0386	1.064	FM
$RbNbTi_2Bi_5$	-53.8134	-53.8134	-53.8134	0	NM
$RbVTi_2Bi_5$	-52.3882	-52.3878	-52.3886	0	NM
$RbZrTi_2Bi_5$	-52.6589	-52.6589	-52.6589	0	NM
$RbTi_3Bi_4Pb$	-51.1371	-51.1371	-51.1371	0	NM
$RbTi_3PbBi_4$	-51.0645	-51.0645	-51.0645	0	NM
$RbTi_3Bi_4Sb$	-52.2005	-52.2005	-52.2005	0	NM
$RbTi_3SbBi_4$	-51.8291	-51.8291	-51.8291	0	NM
$RbTi_3Bi_4Sn$	-51.2928	-51.2928	-51.2928	0	NM
$RbTi_3SnBi_4$	-50.9113	-50.9113	-50.9113	0	NM
$RbTi_3Bi_4Te$	-51.6223	-51.6223	-51.6223	0	NM
$RbTi_3TeBi_4$	-51.5545	-51.5545	-51.5545	0	NM
$RbTi_3Bi_4Ga$	-49.8347	-49.8713	-49.8296	0.937	FM
$RbTi_3GaBi_4$	-49.6967	-49.6967	-49.6967	0	NM
$RbTi_3Bi_3Pb_2$	-50.2968	-50.2597	-50.2962	0	NM
$RbTi_3Bi_3Sn_2$	-50.3297	-50.329	-50.3311	0	NM

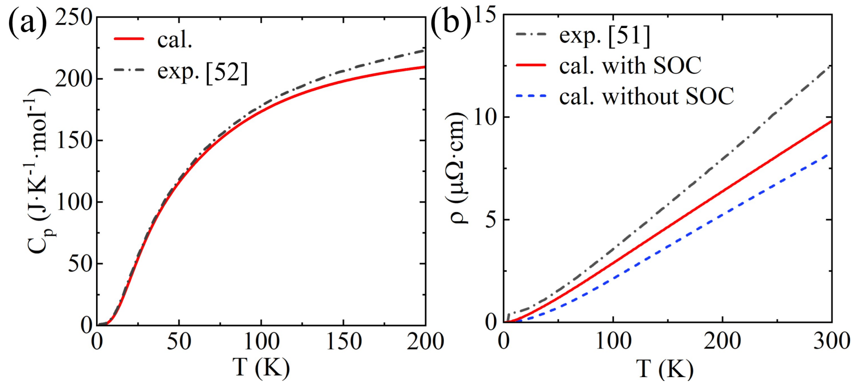

FIG. S3. Heat capacity and transport properties of ATi_3Bi_5. (a) Calculated temperature dependence of the heat capacity Cp of $RbTi_3Bi_5$ in comparison to the experiment [52]. (b) Calculated temperature dependence of the longitudinal electrical resistivity for $CsTi_3Bi_5$ with and without spin-orbit coupling (SOC) compared with the experiment [51].

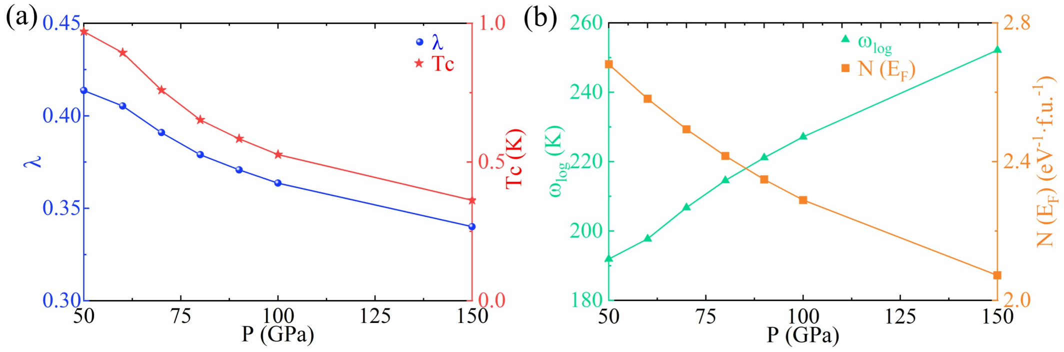

FIG. S4. Superconducting Tc and λ under pressure ranging from 50 to 150GPa. (b) ω_{log} and N(E_F) under pressure ranging from 50 to 150GPa

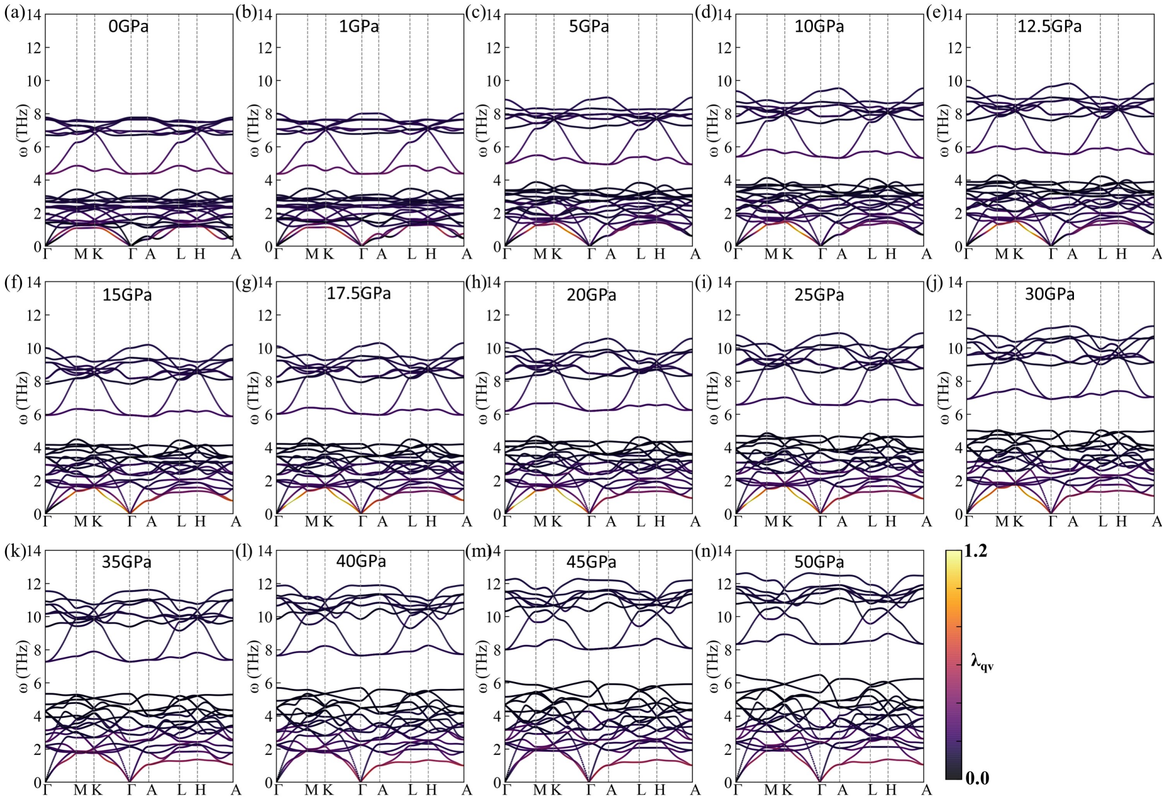

FIG. S5. Superconducting properties of $CsTi_3Bi_5$ under pressure within the range of 0-50GPa. The phonon spectra weighted by EPC strength λ_{qv}, projected phDOS, Eliashberg spectral function $\alpha^2F(\omega)$, and cumulative frequency-dependent EPC $\lambda(\omega)$ at (a) 0, (b) 1, (c) 5, (d) 10, (e) 12.5, (f) 15, (g) 17.5, (h) 20, (i) 25, (j) 30, (k) 35, (l) 40, (m) 45, and (n) 50GPa, respectively. Colorbar represents the value of λ_{qv}.

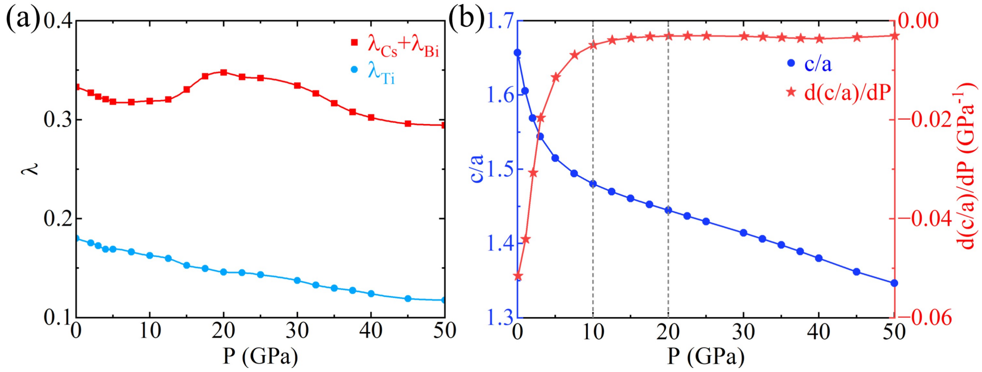

FIG. S6. Divided λ and ratio of lattice parameters c/a under pressure. (a) λ_{Cs+Bi} and λ_{Ti} under pressure in the range of 0-50GPa. (b) The ratio of out-of-plane and in-plane lattice parameters c/a under pressure in the range of 0-50GPa.

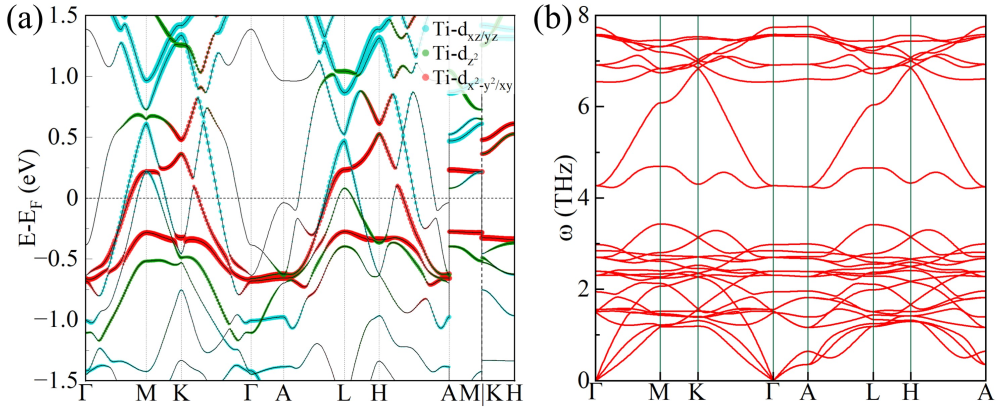

FIG. S7. Electronic band structure and phonon spectrum of $RbTi_3Bi_5$.

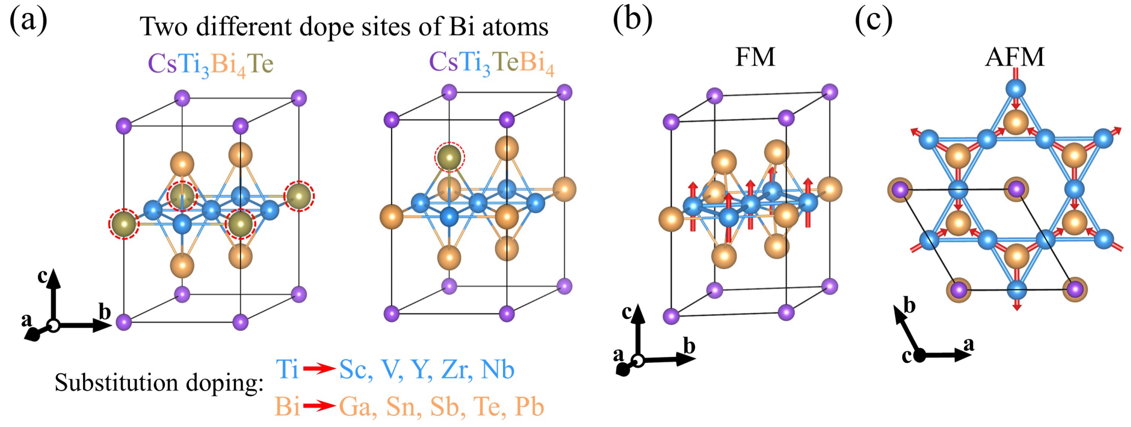

FIG. S8. Doping sites of Bi atoms and ferromagnetic (FM) and antiferromagnetic (AFM) configurations for ATi_3Bi_5 and doped structures. (a) Two different doping sites of Bi atoms. We denote the substitution structure involving Bi2 as $RbTi_3TeBi_4$, and the substitution structure involving Bi1 as $RbTi_3Bi_4Te$. (b) FM and AFM configurations for ATi_3Bi_5 and doped structures.

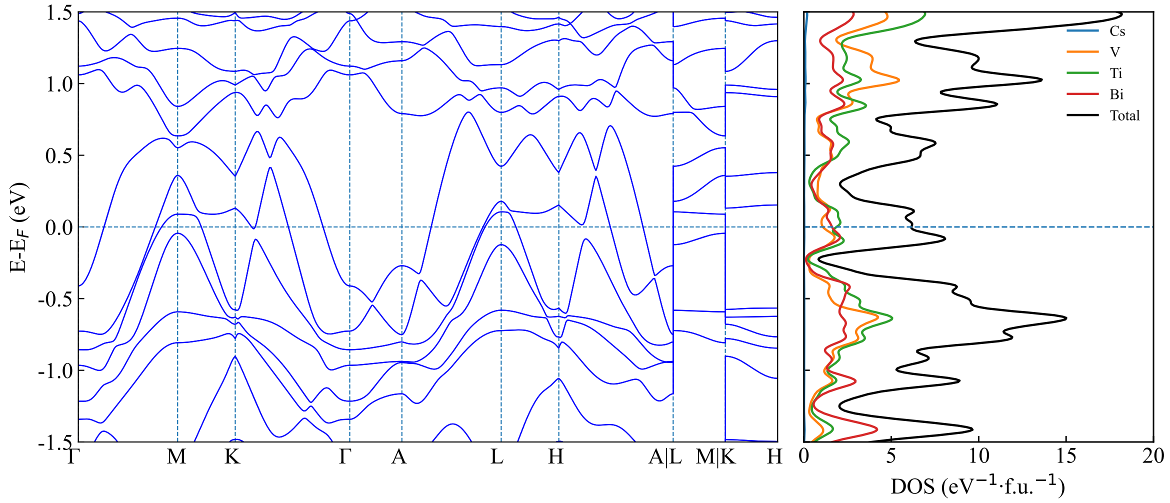

FIG. S9. Electronic band structure and DOS with SOC for $CsVTi_2Bi_5$.

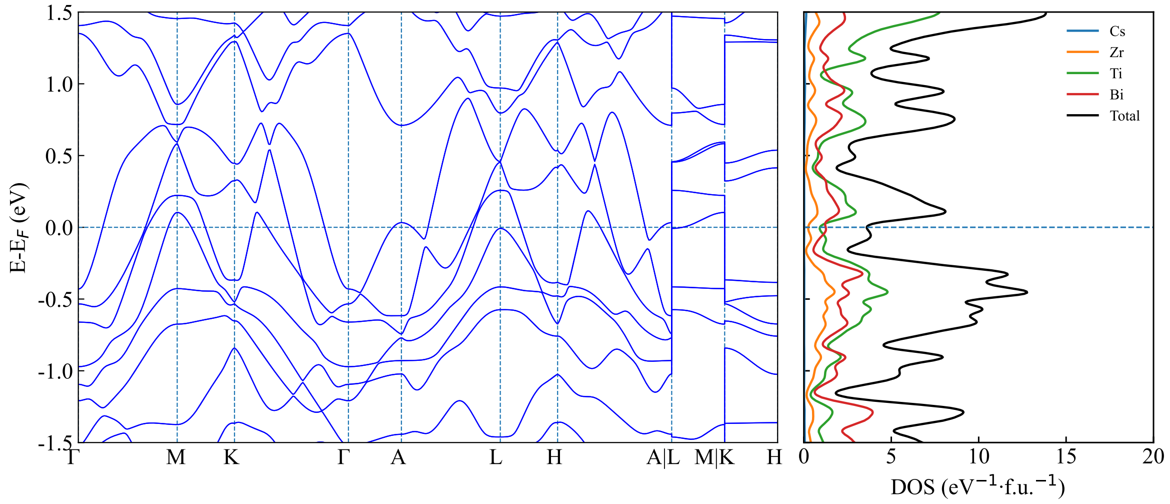

FIG. S10. Electronic band structure and DOS with SOC for $CsZrTi_2Bi_5$.

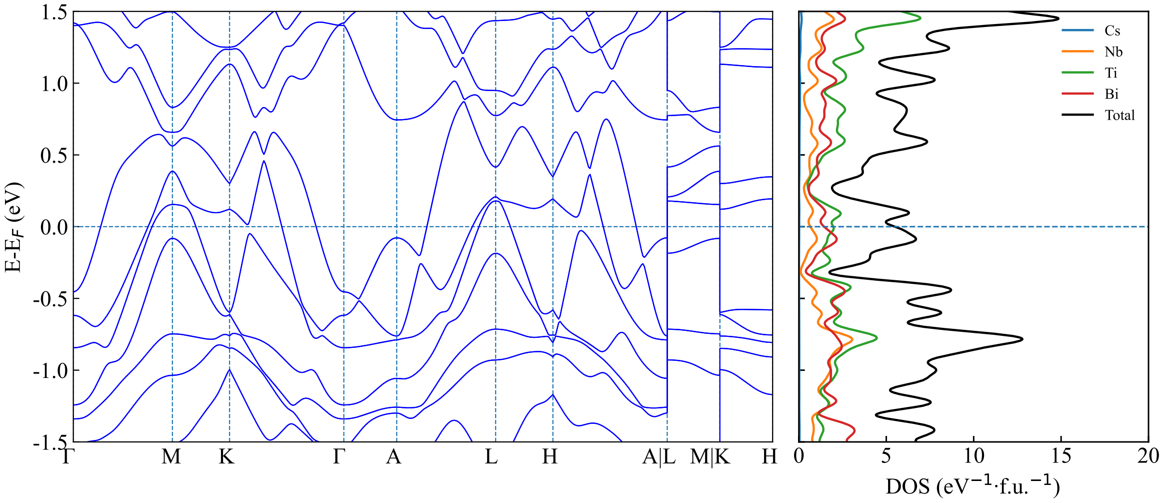

FIG. S11. Electronic band structure and DOS with SOC for $CsNbTi_2Bi_5$.

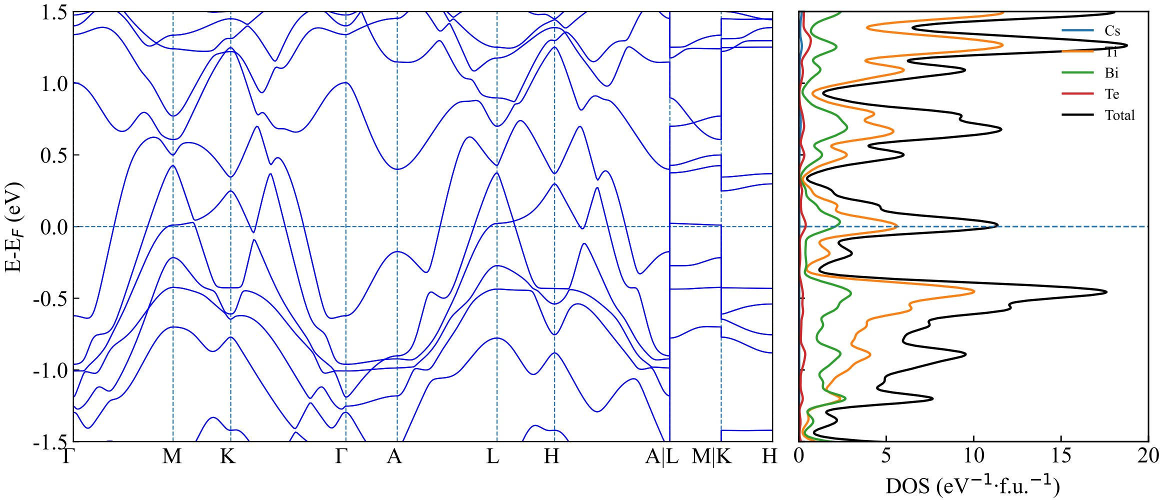

FIG. S12. Electronic band structure and DOS with SOC for $CsTi_3Bi_4Te$.

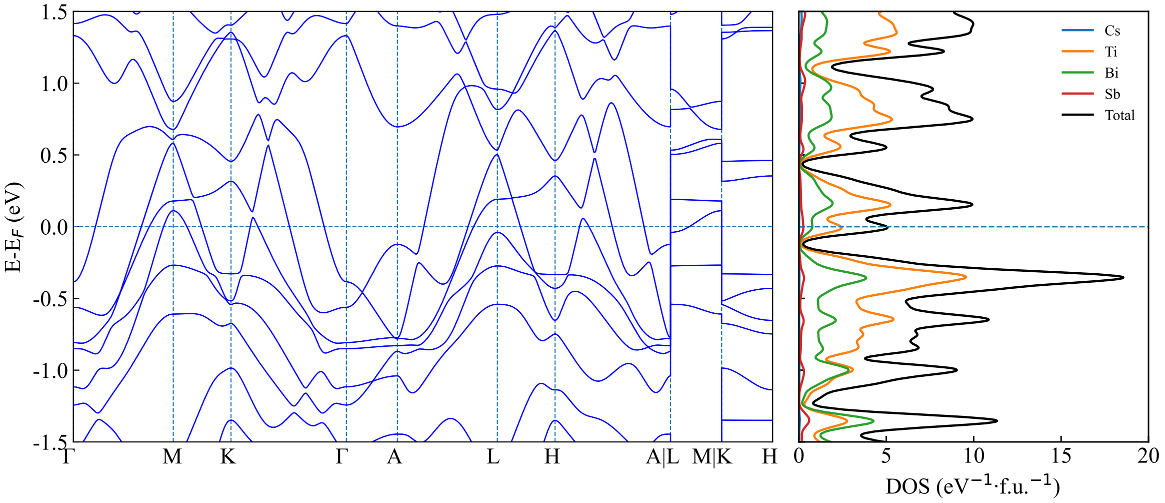

FIG. S13. Electronic band structure and DOS with SOC for $CsTi_3Bi_4Sb$.

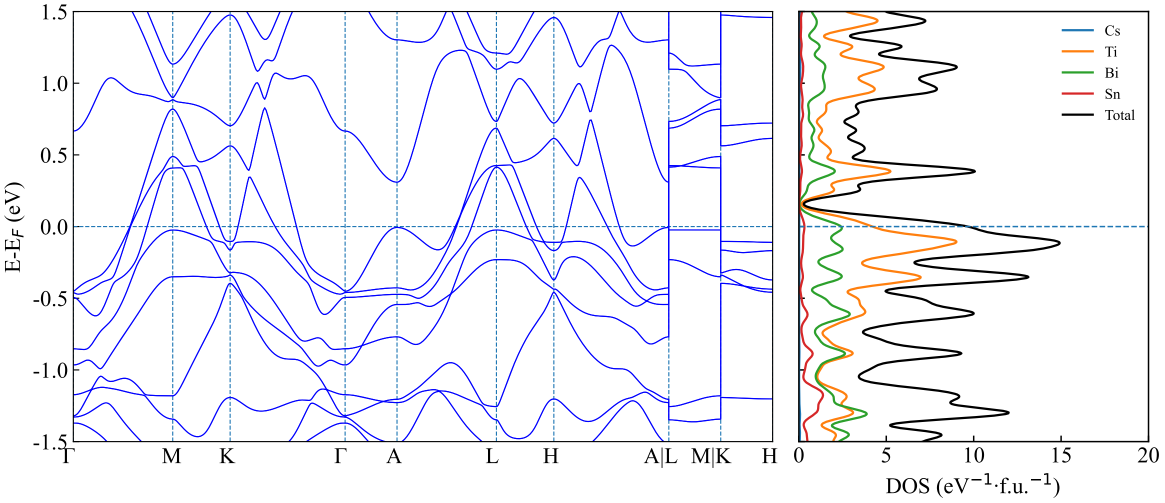

FIG. S14. Electronic band structure and DOS with SOC for $CsTi_3Bi_4Sn$.

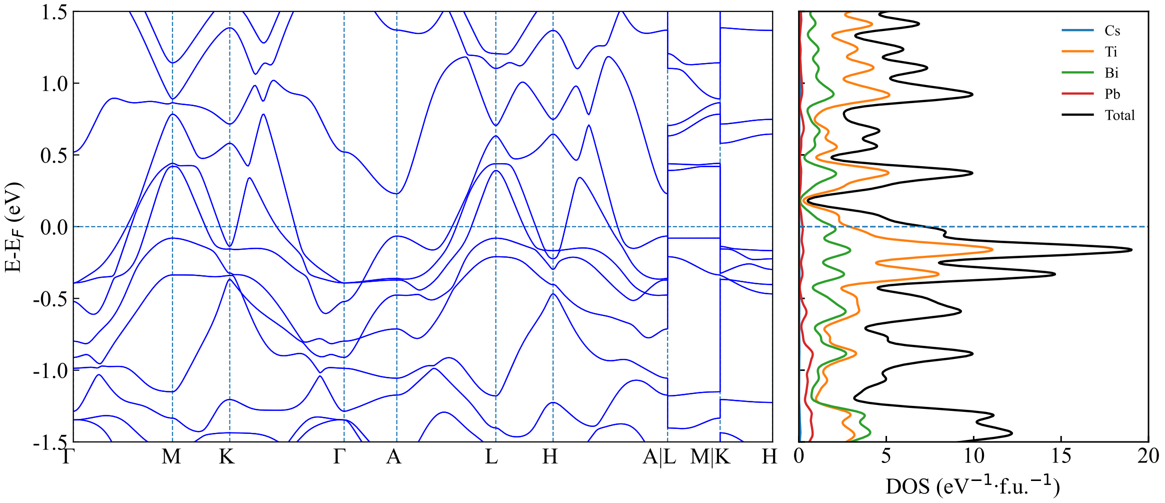

FIG. S15. Electronic band structure and DOS with SOC for $CsTi_3Bi_4Pb$.

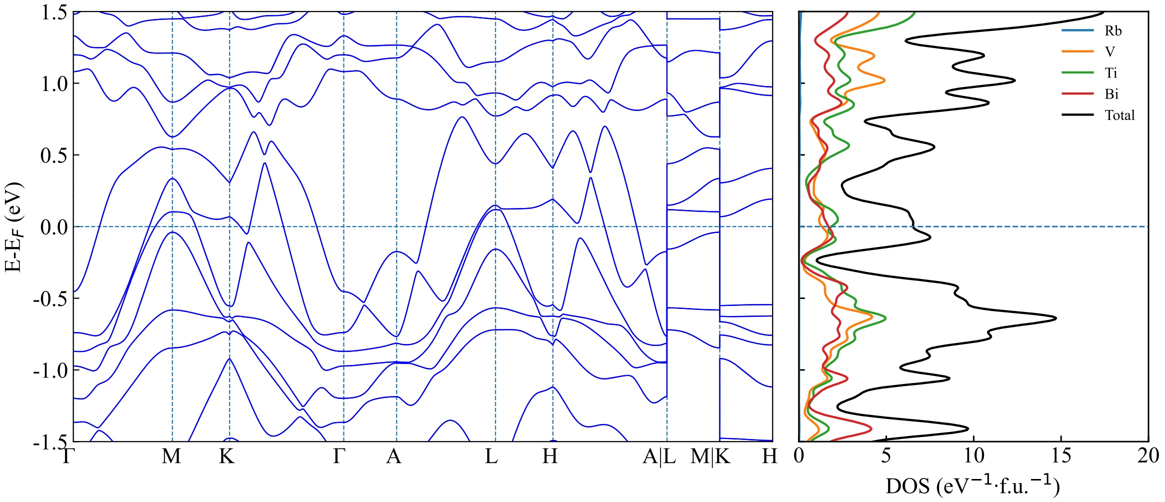

FIG. S16. Electronic band structure and DOS with SOC for $RbVTi_2Bi_5$.

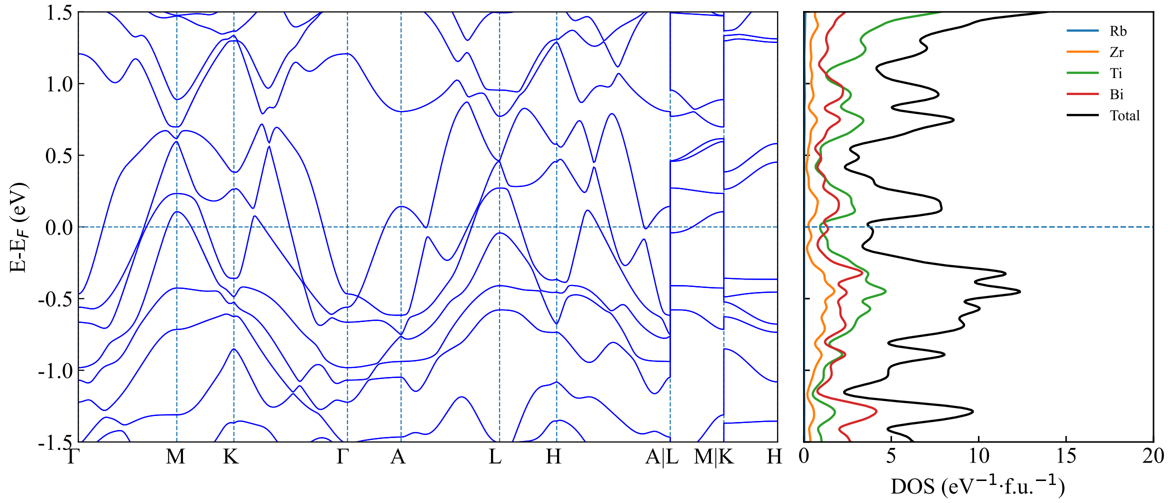

FIG. S17. Electronic band structure and DOS with SOC for $RbZrTi_2Bi_5$.

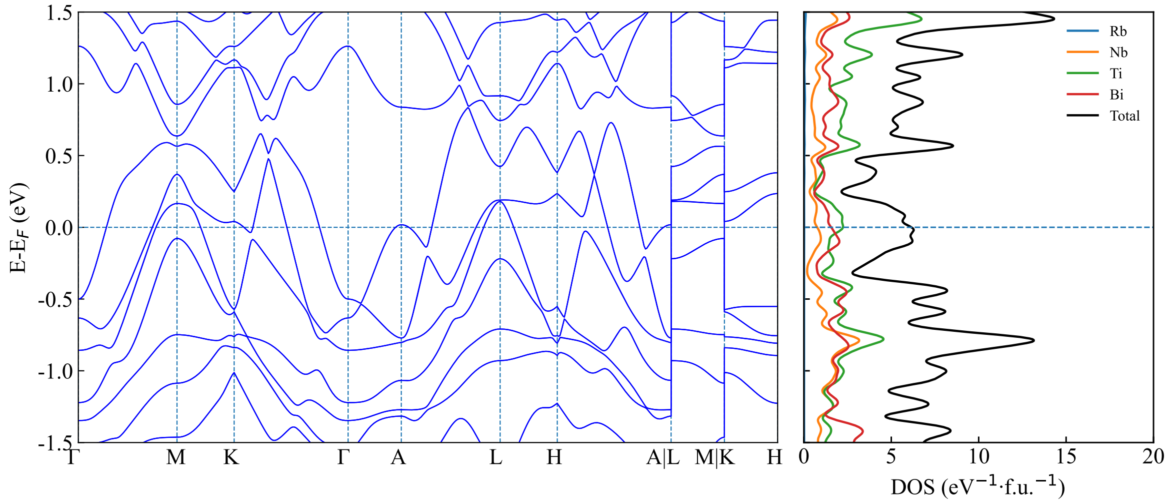

FIG. S18. Electronic band structure and DOS with SOC for $RbNbTi_2Bi_5$.

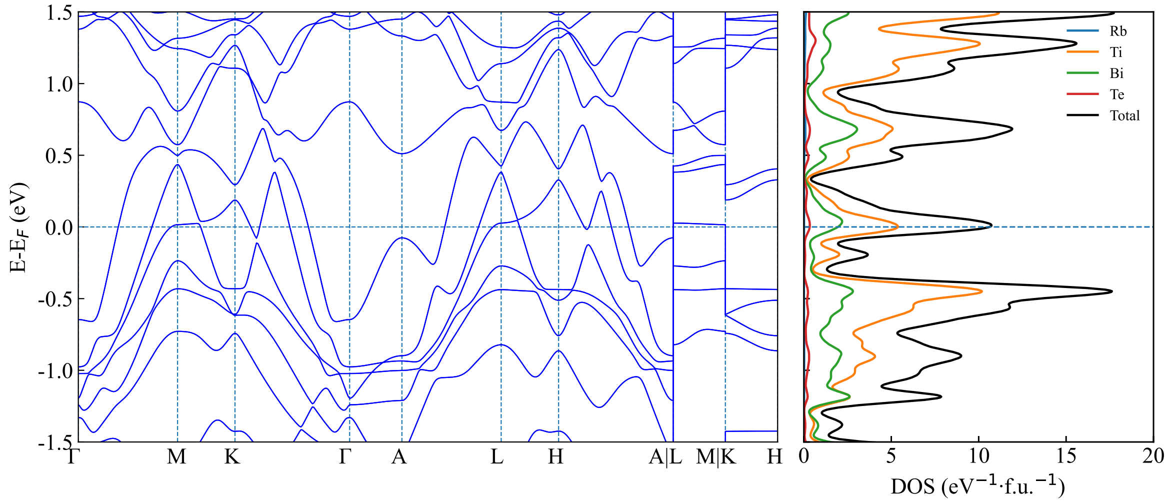

FIG. S19. Electronic band structure and DOS with SOC for $RbTi_3Bi_4Te$.

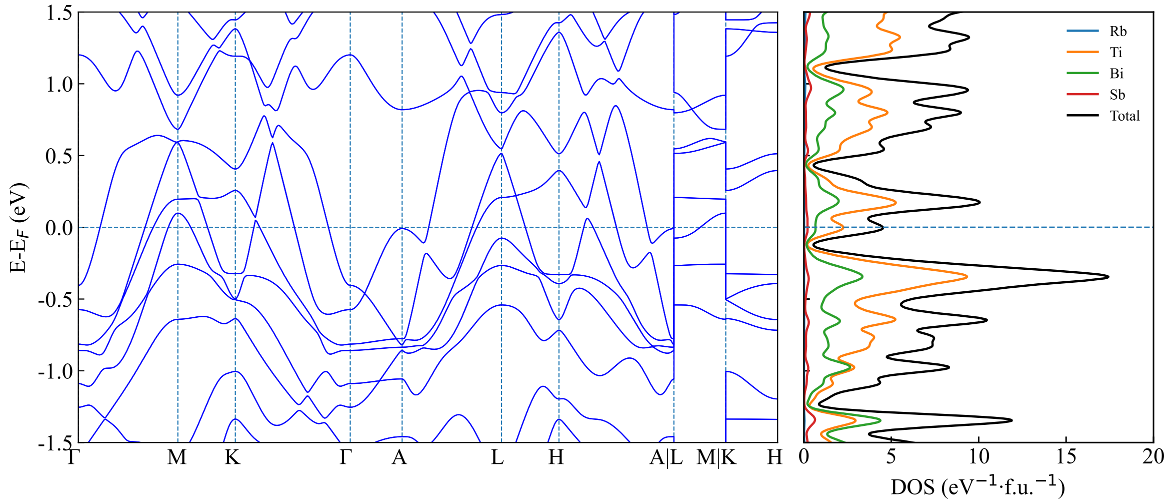

FIG. S20. Electronic band structure and DOS with SOC for $RbTi_3Bi_4Sb$.

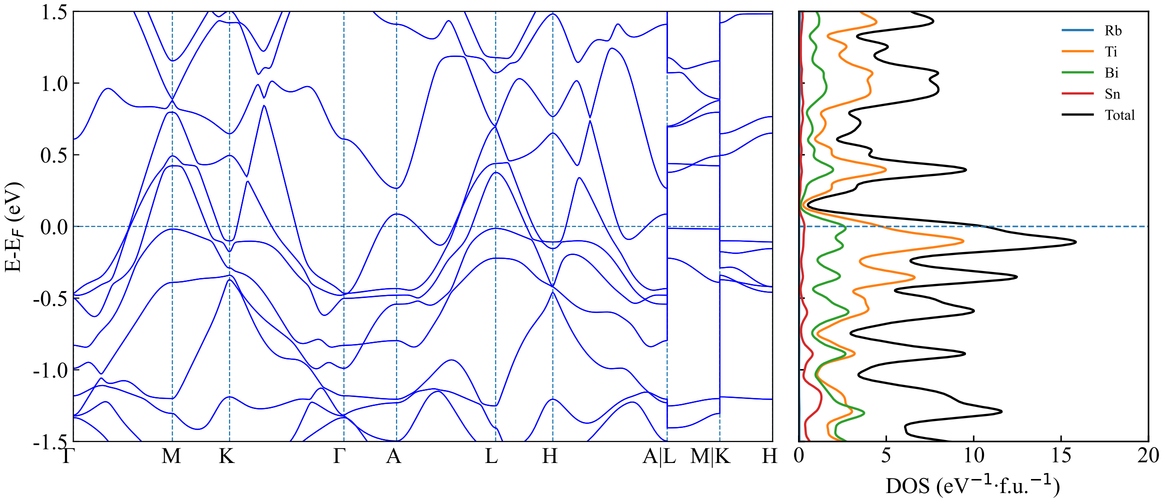

FIG. S21. Electronic band structure and DOS with SOC for $RbTi_3Bi_4Sn$.

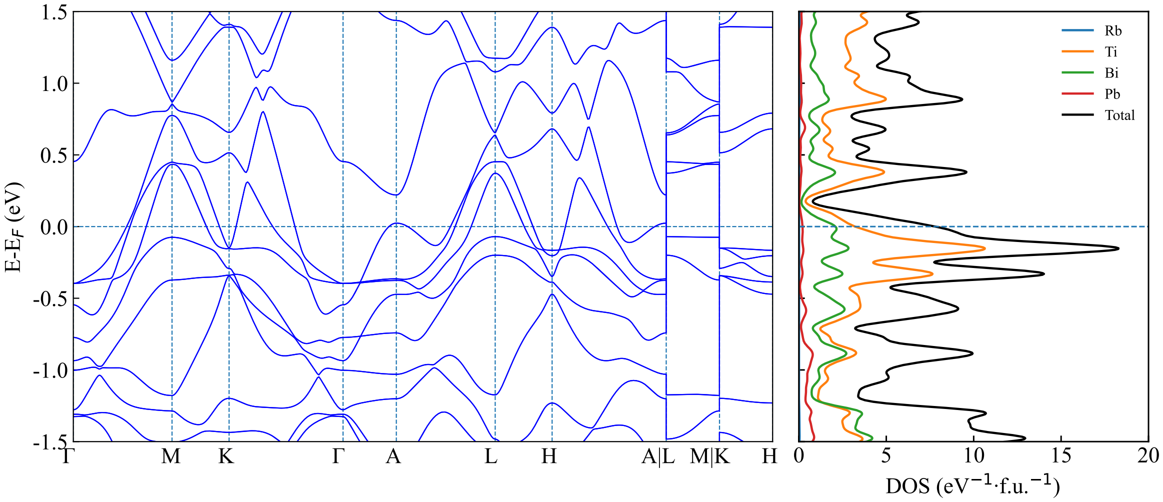

FIG. S22. Electronic band structure and DOS with SOC for $RbTi_3Bi_4Pb$.

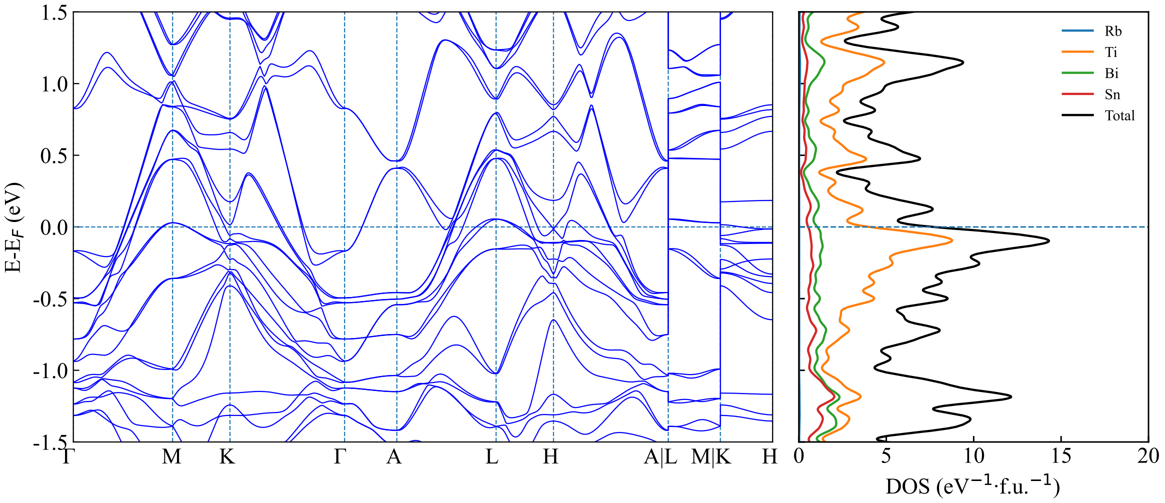

FIG. S23. Electronic band structure and DOS with SOC for $RbTi_3Bi_3Sn_2$.

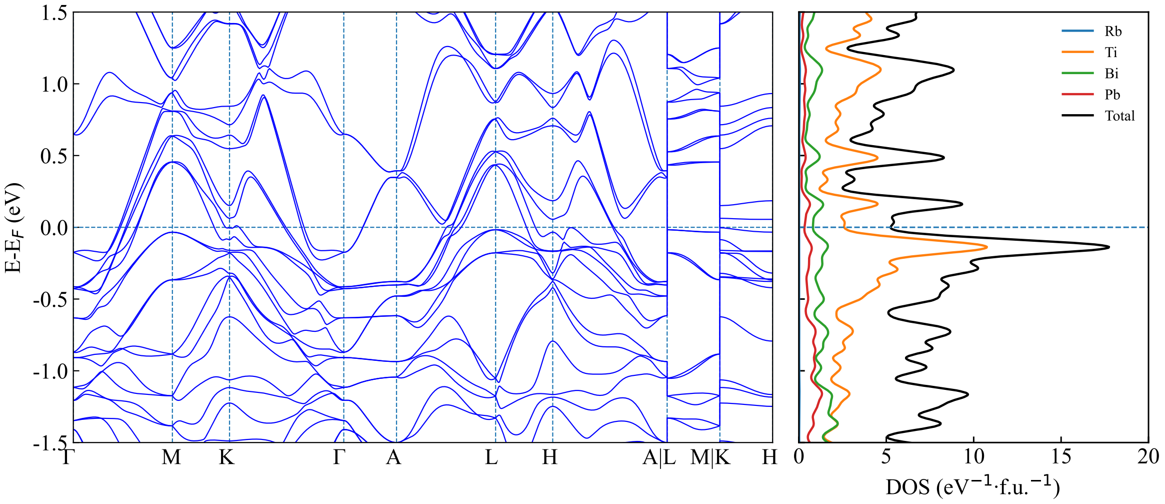

FIG. S24. Electronic band structure and DOS with SOC for $RbTi_3Bi_3Pb_2$.

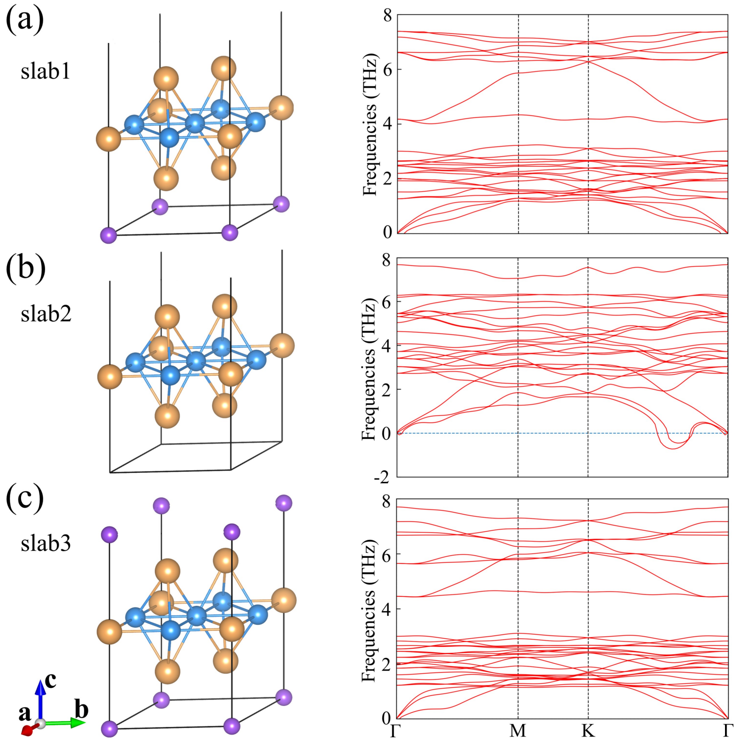

FIG. S25. Three slab structures of $CsTi_3Bi_5$ and their phonon spectra.

TABLE S2. Electronic density of states at the Fermi energy N(E_F) ($eV^{-1}f.u.^{-1}$), logarithmic average frequency $\omega_{\log}$ (K), EPC $\lambda(\omega=\infty)$ and T_c of two stable slab structures of $CsTi_3Bi_5$.

structure	λ	N(E_F) ($eV^{-1}f.u.^{-1}$)	$\omega_{\log}$ (K)	T_c (K)
slab1	0.517	3.789	126.5	1.75
slab3	0.494	4.109	119.7	1.38
